# Spatially‐nested topologies stabilize meta‐ecosystems via cross‐scale source‐sink dynamics

**DOI:** 10.1002/ecy.70375

**Published:** 2026-04-12

**Authors:** Tianna Peller, Isabelle Gounand, Marie‐Josée Fortin, Frédéric Guichard

**Affiliations:** ^1^ Department of Ecology and Evolutionary Biology University of Toronto Toronto Ontario Canada; ^2^ Sorbonne Université, CNRS, UPEC, CNRS, IRD, INRAE Institut d'écologie et des sciences de l'environnement, IEES Paris France; ^3^ Department of Biology McGill University Montréal Québec Canada

**Keywords:** bottom‐up, cross‐ecosystem, cross‐habitat, dispersal, ecosystem functions, food‐web resource flows, spatial networks, spatial subsidies, top‐down

## Abstract

Ecosystems are open to spatial flows of nonliving resources and dispersing organisms that can interact to drive their dynamics and functions. Empirical evidence shows resource flows and dispersal commonly have contrasting properties: resource flows connect nearby ecosystems of different types, while dispersal connects relatively distant ecosystems of similar types. For instance, islands and adjacent coral reefs are connected through resource exchanges but also exhibit organisms that disperse across larger spatial scales between homologous ecosystems. These contrasting properties of spatial flows can yield spatially nested topologies of diverse, coupled ecosystems, where nearby ecosystems coupled via resource flows are embedded within larger scale dispersal networks. Using meta‐ecosystem models, we show that spatially nested topologies of coupled ecosystems have a general stabilizing effect on ecosystem dynamics. Stabilization results from the emergence of cross‐scale source‐sink dynamics, where some ecosystems act as nutrient sources but consumer sinks and others as nutrient sinks but consumer sources—creating a self‐regulating spatial structure that dampens local instabilities. These dynamics lead to spatial variation in trophic regulation and biomass stocks across ecosystems of the same type: consumer sinks exhibit stronger top‐down control and lower primary producer stocks, while consumer sources exhibit stronger bottom‐up control and higher producer stocks. These local effects of cross‐scale source‐sink dynamics scale up to influence functions at the meta‐ecosystem scale, including increasing primary production and nutrient retention. Critically, we further demonstrate how these source‐sink dynamics depend on the dispersal rates of consumer species in both ecosystem types, such that the stability and function of one ecosystem type can be shaped by the dispersal rate of consumers in another. Our findings suggest that the diversity of ecosystem types and the hierarchy of spatial flow scales observed ubiquitously in nature are key properties of spatial ecological systems, driving their stability and functions across scales.

## INTRODUCTION

Spatial flows of nonliving resources (nutrients and detritus) and organism dispersal—the one‐way movement of organisms away from their birthplace (Clobert et al., 2012)—are ubiquitous in nature, and both can have substantial effects on ecological systems. Following a long‐standing divide between community and ecosystem ecology (Massol et al., 2011), however, the impacts of resource flows and dispersal have traditionally been studied separately. Population and community ecology have shown a fundamental role of organism dispersal in maintaining populations with negative growth rates (Amezcua & Holyoak, 2000), stabilizing community dynamics (Briggs & Hoopes, 2004), and regulating community structure (Chase et al., 2010). Separately, ecosystem ecology has demonstrated that resource flows can influence ecosystem stability (Jones & Lennon, 2015), regulate food‐web structure (Allen & Wesner, 2016), and modify the functions ecosystems carry out (Sato et al., 2021). Collectively, this research has established a solid mechanistic understanding of the impacts that resource flows and dispersal can individually have on ecological systems across scales.

In nature, however, resource flows and dispersal rarely occur in isolation (Gounand et al., 2018). Rather, ecosystems typically receive and export resources while also exchanging dispersing organisms with other ecosystems (Menge et al., 2015; Polis et al., 1997). Empirical findings have led to growing recognition that resource flows and dispersal may interact to shape ecological dynamics (Benkwitt et al., 2021; Massol et al., 2011; Richardson & Sato, 2014). In particular, experiments have shown that dispersal is often context‐dependent—its direction, magnitude, and ecological effects can vary with ecosystem properties such as resource availability or population size (Fronhofer et al., 2018). These ecosystem properties can further be affected by resource flows across ecosystem boundaries, as observed in a variety of systems, from coral reefs and intertidal zones (Graham et al., 2018; Hyndes et al., 2022) to ponds and lakes (Cottingham & Narayan, 2013; Earl & Semlitsch, 2011). For example, studies on coral reefs have shown how resource flows from adjacent island ecosystems can enhance the biomass of herbivorous fish (Graham et al., 2018), just as flows of leaf detritus from forests to ponds have been observed to enhance zooplankton abundance (Cottingham & Narayan, 2013). To better understand their interaction, meta‐ecosystem theory has emerged as a framework that explicitly integrates resource flows and organism dispersal, demonstrating that their interaction can drive both local and regional dynamics (Gounand et al., 2018; Harvey et al., 2020). For instance, when ecosystems are coupled via resource flows and dispersal, theory predicts that net resource flows and dispersal can emerge in opposite directions between ecosystems, creating strong interdependencies (Gravel et al., 2010). Altogether, these studies suggest that understanding the effects of resource flows and dispersal on ecological systems, as well as their importance for achieving conservation objectives (Carr et al., 2017), requires explicit consideration of their interaction.

A key challenge of making general predictions on interactions between resource flows and dispersal is that they can form complex spatial networks of numerous coupled ecosystems, directly and indirectly interacting across landscapes. Faced with understanding a bewildering number of interacting elements, an approach that has proven invaluable in ecology is the study of subsets of interacting elements (i.e., “motifs”) that are frequently observed within larger, complex networks (McCann, 2012). In particular, food‐web ecology has thoroughly developed this approach, identifying patterns of trophic interactions between species (e.g., apparent competition, omnivory) that frequently occur within real‐world food‐webs due to constraints, such as body size (Stouffer & Bascompte, 2010), or their dynamical importance (Borrelli, 2015). By providing a tractable unit of investigation, the study of food‐web motifs has revealed fundamental effects of species diversity and enabled a mechanistic understanding of how the structure of interactions between species governs stability. Ultimately, to develop a similar mechanistic understanding of the significance of interactions between ecosystems, we need to identify constraints on the topology of different types of spatial flows and the frequent patterns of interactions between ecosystems that they yield (herein, “meta‐ecosystem motif(s)”).

**FIGURE 1:**
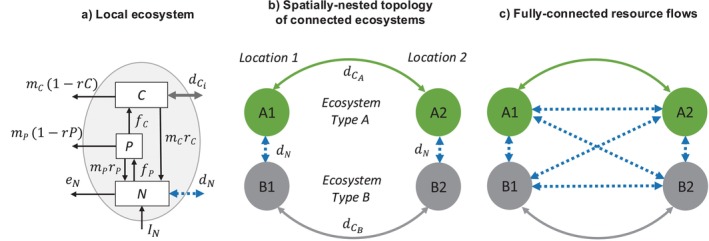
Conceptual diagram of the meta‐ecosystem model. (a) In each of the four local ecosystems, inorganic nutrients *N* are taken up by primary producers *P*, which are grazed on by consumers *C* according to Monod‐type uptake functions *f*_*P*_ and *f*_*C*_. *P* and *C* are subject to losses at respective rates *m*_*P*_ and *m*_*C*_, a fraction of which, *r*_*P*_ and *r*_*C*_, is contributed to the inorganic nutrient pool. *N* receives constant nutrient input *I*_*N*_ and exports nutrients at rate *e*_*N*_. Ecosystems are connected via resource flows at rate *d*_*N*_ (blue dashed arrow) and/or dispersal at rate *d*_*Ci*_ (gray arrow; ecosystem‐specific colors shown in panels (b) and (c)) between homologous ecosystem compartments. (b) and (c): The meta‐ecosystem consists of two ecosystem types. Type A (green) and Type B (gray), each exhibiting ecosystem‐specific dispersal rates $d_{C_{A}}$ and $d_{C_{B}}$, respectively. In (b)—spatially nested topology of connected ecosystems—dispersal connects ecosystems of the same type across larger distances relative to resource flows, which connect nearby ecosystems of different types, whereas in (c) —the meta‐ecosystem with fully connected resource flows—all ecosystems are connected by resource flows, regardless of distance.

Mounting evidence indicates that resource flows and dispersal commonly couple ecosystems according to contrasting characteristics (Gounand et al., 2018). Whereas resource flows commonly couple nearby ecosystems of different types (Graham et al., 2018; Muehlbauer et al., 2014), the dispersal of many species couples similar ecosystems across relatively large distances (Peller et al., 2021; Shanks, 2020). This can yield a spatially nested topology of connected ecosystems (Figure 1), whereby cross‐ecosystem resource flows between nearby ecosystems are embedded within larger scale dispersal networks. For instance, terrestrial and freshwater ecosystems exchange resources at their boundary (Peller & Altermatt, 2024; Bartels et al., 2012) while exhibiting independent dispersal processes connecting them to similar ecosystems across larger scales (Havel & Shurin, 2004). Kelp forests exchange inorganic nutrients and detritus with adjacent intertidal ecosystems (Dugan et al., 2011), and both types of ecosystems exhibit organisms that can disperse across larger spatial scales along the coast (Anadón et al., 2013). Similarly, islands and adjacent marine ecosystems, such as coral reefs, are connected through resource exchanges (Graham et al., 2018) but also exhibit organisms that disperse across larger spatial scales between homologous ecosystems (Gillespie et al., 2012). Notably, such observations have led to predictions that resource flows may have consequences beyond their spatial scale by determining the source or sink status of dispersing organisms to more distant ecosystems (Benkwitt et al., 2021). However, despite these real‐world examples, meta‐ecosystem theory typically overlooks the contrasting characteristics of resource flows and dispersal and the spatially nested topologies of coupled ecosystems they yield. Consequently, we lack an understanding of how multi‐scale interactions between resource flows and dispersal influence ecosystem dynamics and functions.

Here, we study the spatially nested topology of coupled ecosystems—where resource flows couple different ecosystem types across smaller distances than organism dispersal—as a meta‐ecosystem motif: a building block of spatial networks of diverse ecosystems coupled by different types of spatial flows. We investigate the dynamics, trophic regulation, and functions of meta‐ecosystems with spatially nested topologies of coupled ecosystems compared to a control topology, where resource flows are not restricted by distance. We show that spatially nested topologies allow for the emergence of cross‐scale source‐sink dynamics that have a general stabilizing effect on meta‐ecosystem dynamics. The emergent source‐sink dynamics drive spatially heterogeneous trophic regulation and ecosystem functions and scale up to influence meta‐ecosystem functions. Our findings demonstrate the diversity of ecosystem types and the spatial hierarchy of spatial flows observed in nature as key drivers of the stability and functions of ecosystems across scales. Analogous to food‐web motifs of diverse interacting species, our study further shows that a motif perspective can provide fundamental insights into how the spatial structure of interactions between diverse sets of ecosystems governs ecological properties.

## MODEL AND METHODS

### Meta‐ecosystem model

We focus our analysis on a general meta‐ecosystem model with four local ecosystems (Figure 1) belonging to two distinct ecosystem types: Type A and Type B. Each local ecosystem has an inorganic nutrient (*N*), primary producer (*P*), and consumer (*C*) compartment (Figure 1a). Primary producers take up nutrients for growth and are grazed on by consumers. We use saturating Monod‐type uptake functions for the primary producer (*f*_*P*_) and the consumer (*f*_*C*_), with maximum uptake rate *a*_*x*_ and half‐saturation constant *b*_*x*_ for each trophic level *x*. Primary producers and consumers are lost through mortality and other factors at rates *m*_*p*_ and *m*_*c*_, respectively. A fraction of nutrients released through producer and consumer losses, *r*_*p*_ and *r*_*c*_, is recycled within the local ecosystem, and the remainder is lost from the meta‐ecosystem. Local ecosystems are open at the inorganic nutrient level: they receive a constant and independent input of inorganic nutrients *I*_*N*_ and export nutrients at constant rate $e_{N}$. Parameter values are identical across the four local ecosystems (see Appendix S1: Table S1 for a summary of state variables and parameters). The dynamics of the meta‐ecosystem are given by the following set of equations, with *i* denoting the ecosystem:

(1) $$\frac{dN_{i}}{\mathrm{dt}}=I_{N}-e_{N}N_{i}+m_{P}r_{P}P_{i}+m_{C}r_{C}C_{i}-P_{i}f_{P_{i}}\left(N_{i}\right)+d_{N}\sum_{j=1}^{n}s_{N_{ij}}N_{j},$$

(2) $$\frac{dP_{i}}{\mathrm{dt}}=P_{i}f_{P_{i}}\left(N_{i}\right)-m_{P}P_{i}-C_{i}f_{C_{i}}\left(P_{i}\right),$$

(3) $$\frac{dC_{i}}{\mathrm{dt}}=C_{i}f_{C_{i}}\left(P_{i}\right)-m_{C}C_{i}+d_{C_{i}}\sum_{j=1}^{n}s_{C_{ij}}C_{j},$$

(4) $$f_{P}\left(N_{i}\right)=\frac{a_{P}N_{i}}{b_{P}+N_{i}},$$

(5) $$f_{C}\left(P_{i}\right)=\frac{a_{C}P_{i}}{b_{C}+P_{i}}.$$

The meta‐ecosystem exhibits a spatially nested topology of coupled ecosystems consisting of two distant locations (Location 1 and Location 2; Figure 1b). Each location contains two adjacent local ecosystems—one ecosystem of Type A and one of Type B (Figure 1b, different colors). Within each location, Type A and Type B ecosystems are coupled by resource flows, specifically flows of inorganic nutrients, at rate *d*_*N*_ (dashed arrows), whereas distant locations are connected by consumer dispersal between ecosystems of the same type at rate $d_{C_{i}}$ (i.e., Type A Location 1–Type A Location 2; Type B Location 1–Type B Location 2; solid arrows). Type A and Type B ecosystems have independent dispersal rates $d_{C_{A}}$ and $d_{C_{B}}$. We assume that primary producer dispersal occurs at the within‐ecosystem scale (e.g., Kinlan & Gaines, 2003), and thus does not couple ecosystems at distant locations (but see Appendix S1: Section S2, where we relax this assumption). Spatial flows of each compartment *x =* {*N,P,C*} are bidirectional and implemented in connectivity matrices *S*_*x*_ (Jansen & Lloyd, 2000). Each connectivity matrix is an *n × n* matrix, where *n* = 4 is the number of ecosystems in the meta‐ecosystem; off‐diagonal entries (*s*_*ij*_ ≥0, *i ≠ j*) represent spatial flows entering ecosystem *j* from ecosystem *i* for ecosystem compartment *x*; and diagonal entries (*s*_*ii*_ < 0) represent spatial flows leaving ecosystem *i* through ecosystem compartment *x*. We control for the effects of the spatially nested meta‐ecosystem topology by analyzing a model that is identical in all respects, except that it exhibits fully connected resource flows, that is, resource flows connect each pair of ecosystems in the meta‐ecosystem (Figure 1c).

### Analysis

We use numerical simulations to study the meta‐ecosystem, using solvers ode45 and ode15s in MATLAB R2022b (Mathworks, 2022).

#### Stable states

We simulate the dynamics of the meta‐ecosystem across a range of resource flow rates coupling ecosystems of different types, from no resource flow to high rates of resource flow. For each resource flow rate, we simulate the dynamics across a range of dispersal rates coupling ecosystems of the same type. For each combination of resource flow and dispersal rates, we keep all other parameter values constant and simulate the dynamics for 50 sets of initial conditions (see Appendix S1: Section S3 for an expanded analysis) to identify the stable states of the meta‐ecosystem. We determine the stability of a state based on the dynamics consistently converging to and remaining at the same point or cyclic attractor across sets of initial conditions.

#### Contrasting spatial scales of resource flows and dispersal

The meta‐ecosystem with the spatially nested topology of connected ecosystems captures, implicitly, that dispersal couples ecosystems across greater spatial scales than resource flows by assuming there are no resource flow connections between ecosystems coupled by dispersal (Figure 1b). We further integrate differences in the spatial scales of resource flows and dispersal into our analysis by recognizing the rate of exchange between two ecosystems can decrease with the distance between them (Muehlbauer et al., 2014). Studies suggest that dispersal can connect ecosystems across scales that are an order of magnitude greater than the scale of resource flows across ecosystems (Peller et al., 2021). Thus, we study resource flow rates *d*_*N*_ coupling ecosystems at up to an order of magnitude greater than rates of consumer dispersal $d_{C_{i}}$.

#### Net spatial flows, source and sink ecosystems, and trophic regulation

We determine the net direction of resource flows and dispersal and their effect on trophic regulation in each ecosystem *i*. For each ecosystem, we calculate the net resource flows and dispersal, individually, as the difference in the spatial flow terms (Equation 1 for resource flows and Equation 3 for dispersal) into versus out of the ecosystem: $d_{x}\sum_{j=1}^{n}\left(s_{\mathrm{xij}}x_{j}\right).$ We use this metric to determine whether an ecosystem is a source or sink for ecosystem compartment *x* (i.e., inorganic nutrients or consumers), defining source or sink status based on the net direction of spatial flows, not on whether an ecosystem is self‐sustaining (Loreau et al., 2013). A negative value indicates the ecosystem is a net exporter and thus a *source* of that ecosystem compartment, while a positive value indicates the ecosystem is a net importer and a sink of that ecosystem compartment.

Resource flows and consumer dispersal can directly influence the stocks of inorganic nutrients and consumers in the ecosystems, respectively. Consequently, they can affect the trophic regulation of primary producer stocks, which take up nutrients for growth and are consumed by consumers. We quantify the regulatory effect of resource flows and dispersal as their net effect (*E*_*i*_) on the stocks of primary producers in each ecosystem *i*. The net effect is obtained by determining how each process regulating primary producer stocks—the uptake of nutrients by primary producers (bottom‐up regulation) and the consumption of primary producers by consumers (top‐down regulation)—is influenced by spatial flows relative to the other regulating process, using the following equation (following Peller et al., 2022):

(6) $$E_{i}=\left(C_{i}^{d>0}f_{C_{i}}{\left(P_{i}\right)}^{d>0}-C_{i}^{d=0}f_{C_{i}}{\left(P_{i}\right)}^{d=0}\right)-\left(P_{i}^{d>0}f_{P_{i}}{\left(N_{i}\right)}^{d>0}-{P_{i}}^{d=0}f_{P_{i}}{\left(N_{i}\right)}^{d=0}\right).$$

*E*_*i*_ > 0 (*E*_*i*_ < 0) indicates spatial flows strengthen secondary (primary) production relative to primary (secondary) production and thus have a net top‐down (bottom‐up) impact on primary producer stocks of ecosystem *i*. *E*_*i*_ = 0 indicates that any change in secondary production in response to spatial flows is matched by a change in primary production.

#### Local and meta‐ecosystem functions

We assess the implications of the different stable states for ecosystem function at local and meta‐ecosystem scales. We measure local ecosystem function as the long‐term average of primary $P_{i}f_{P_{i}}\left(N_{i}\right)$ and secondary $C_{i}f_{C_{i}}\left(P_{i}\right)$ production, and nutrient retention (as turnover time: total local nutrient stocks (*N + P + C*) divided by the instantaneous rate of local nutrient loss; Massé Jodoin & Guichard, 2019) in each ecosystem *i*. We measure meta‐ecosystem function as the long‐term average of total primary production, secondary production, and nutrient retention for the entire meta‐ecosystem.

#### Ecosystem‐specific dispersal rates

We first carry out all analyses under the assumption that dispersal rates are equal for both ecosystem types ($d_{C_{A}}$ = $d_{C_{B}}$). Different types of ecosystems, however, can be subject to different environmental factors and provide habitat for different organisms, which can result in different rates of organism dispersal for different ecosystem types (Clobert et al., 2012). The structure of our model provides the opportunity to assess whether the dispersal rate of one ecosystem type can influence the dynamics of a different ecosystem type that it exchanges resources with. To explore this, we subsequently repeat all analyses while allowing dispersal rates to differ between the two ecosystem types ($d_{C_{A}}$ ≠ $d_{C_{B}}$).

## RESULTS

### Spatially nested topologies stabilize meta‐ecosystems via cross‐scale source–sink dynamics

**FIGURE 2:**
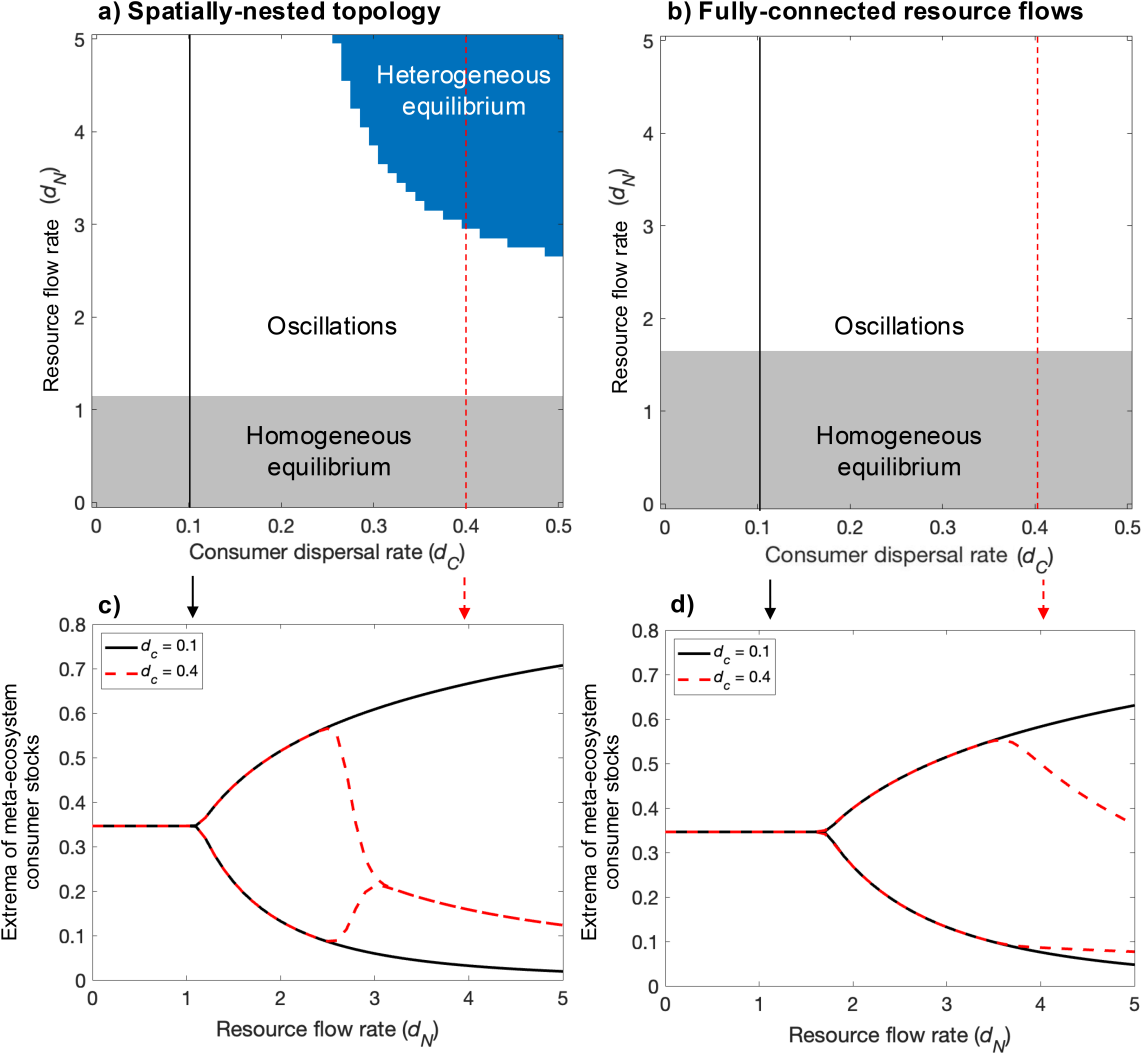
Qualitative states across rates of consumer dispersal *d*_*C*_ and resource flows *d*_*N*_ for meta‐ecosystems with (a) the spatially nested topology of coupled ecosystems and (b) fully connected resource flows. Gray areas indicate a stable homogeneous equilibrium, where ecosystem stocks are identical across local ecosystems; white areas indicate oscillatory dynamics, and blue areas indicate a stable heterogeneous equilibrium, where ecosystem stocks differ across local ecosystems. Corresponding bifurcation diagrams of consumer minimum and maximum stock values at the meta‐ecosystem level for dispersal rates *d*_*C*_ = 0.1 and *d*_*C*_ = 0.4, corresponding to the solid black and red dashed vertical lines in panels (a) and (b) in meta‐ecosystems with (c) the spatially nested topology of coupled ecosystems and (d) fully connected resource flows. In all panels, the dispersal rate is equivalent for both ecosystem types. Parameter values used to generate the figure are *I*_*N*_ = 0.05, *e*_*N*_ = 0.01, *m*_*P*_ = 0.05, *m*_*C*_ = 0.24, *a*_*P*_ = 1, *b*_*P*_ = 10, *a*_*C*_ = 0.6, *b*_*C*_ = 6, *r*_*P*_ = 0.8, and *r*_*C*_ = 0.8.

The spatially nested topology of connected ecosystems yields a unique stable state that is not observed for the fully connected resource flow topology (blue zone, Figure 2). On increasing the rate of resource flows, meta‐ecosystems with both topologies transition from a spatially homogeneous equilibrium to oscillations, regardless of the rate of dispersal. The oscillatory state is predominantly spatially homogeneous but becomes spatially heterogeneous for high rates of resource flows and dispersal (Figure 2c, dashed line: $d_{N}$ = 2.65–2.95; Figure 2d, dashed line: $d_{N}$ > 3.7). For the spatially nested topology only, however, further increasing the rate of resource flows can stabilize the oscillations, resulting in a spatially heterogeneous equilibrium (Figure 2a, blue; Figure 2c, dashed line: $d_{N}$ > 2.95; see Appendix S1: Section S5 for an expanded range of $d_{N}$). The spatially heterogenous equilibrium requires consumer dispersal rates to be sufficiently high and a strong contrast between resource flow and dispersal rates. For the fully connected resource flow topology, the spatially heterogeneous equilibrium is not achieved despite a gradual dampening of the oscillations. In Appendix S1: Section S5, we show that this qualitative difference between the spatially nested topology and the fully connected resource flow topology is not limited to the parameter space explored in the main text. In Appendix S1: Section S6, we show that the predictions are similar in meta‐ecosystems of larger size and with a different dispersal topology.

**FIGURE 3:**
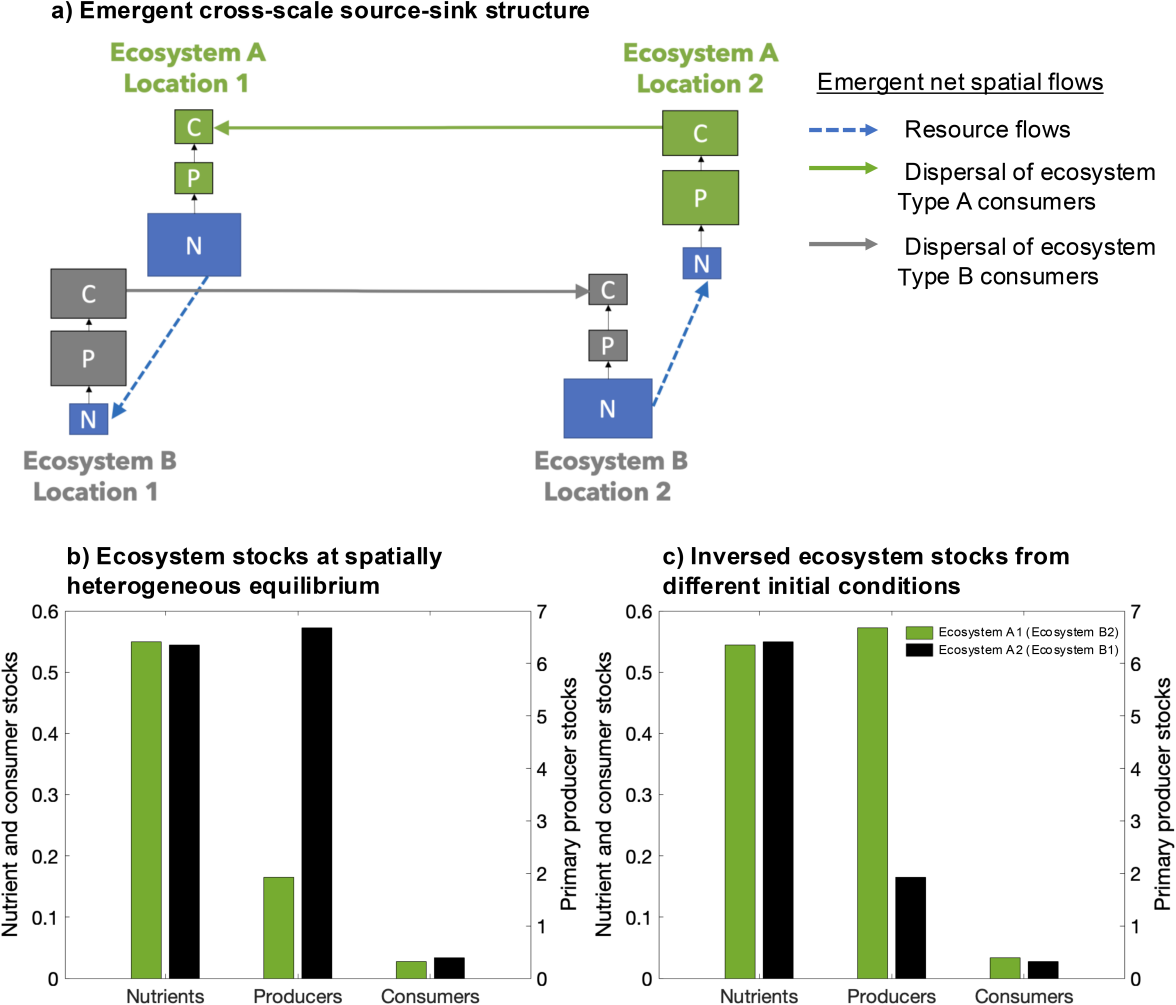
Emergent cross‐scale source‐sink dynamics across ecosystem types yield a spatially heterogeneous equilibrium in spatially nested meta‐ecosystems. (a) Illustration of the cross‐scale source‐sink structure: emergent *net flows* of resources across ecosystem types (blue dashed arrows) allow one ecosystem of each type to become a source of consumer dispersal for the other ecosystem of the same type (Type A: green arrow; Type B: gray arrow) through bottom‐up control. Ecosystems on the receiving end of dispersal are sinks of consumers, which through top‐down regulation, increases inorganic nutrient stocks and leads to these ecosystems being sources (net exporters) of resources for the other ecosystem type. Compartment sizes illustrate source‐sink dynamics but are not proportional to stocks. (b) Local ecosystem stocks for the spatially heterogeneous equilibrium reached when cross‐scale source‐sink dynamics emerge. The pattern of ecosystem stocks is the same for the different ecosystem types due to identical parameterization; each bar represents stocks for an ecosystem compartment of one Type A and one Type B ecosystem (green bars: Type A Location 1 and Type B Location 2; black bars: Type A Location 2 and Type B Location 1). (c) Different sets of initial conditions can invert ecosystem stocks across the meta‐ecosystem. Parameter values used to generate (b) and (c) are *I*_*N*_ = 0.05, *e*_*N*_ = 0.01, *m*_*P*_ = 0.05, *m*_*C*_ = 0.24, *a*_*P*_ = 1, *b*_*P*_ = 10, *a*_*C*_ = 0.6, *b*_*C*_ = 6, *r*_*P*_ = 0.8, *r*_*C*_ = 0.8, *d*_*C*_ *=* 0.4, and *d*_*N*_ = 5. Dispersal rates are equal for both ecosystem types.

The spatially heterogeneous equilibrium observed for the spatially nested topology of coupled ecosystems results from the emergence of cross‐scale source‐sink dynamics (Figure 3a). Specifically, we observe a net flow of inorganic nutrients between different ecosystem types and going in opposite directions in the two locations: a net flow of nutrients from ecosystem Type A to ecosystem Type B in one location and from ecosystem Type B to ecosystem Type A in the other distant location (Figure 3a, blue arrows). Ecosystems that emerge as sinks of inorganic nutrients emerge as sources of consumers dispersing to the distant homologous ecosystem (Figure 3a, gray and green arrows), whereas ecosystems that emerge as sources of inorganic nutrients emerge as sinks of consumer dispersal. We reiterate, for clarity, that the net flows described are an emergent property and not imposed. In contrast, for the fully connected resource flow topology, equilibrium source‐sink dynamics do not emerge. This is because each ecosystem maintains at least one resource flow connection whose net direction fluctuates over time rather than converging to consistent source or sink roles as observed in the spatially nested topology (Appendix S1: Section S7).

### Cross‐scale source‐sink dynamics drive spatially heterogeneous local ecosystem properties

At the spatially heterogeneous equilibrium, local ecosystem stocks differ between ecosystems of the same type (e.g., between ecosystem Type A at Location 1 and Location 2; Figure 3b,c). In particular, primary producer stocks are substantially different, whereas there is a slight difference in nutrient and consumer stocks. This pattern of local ecosystem stocks is identical for both ecosystem types and occurs in an alternating manner across the meta‐ecosystem: stocks in ecosystem Type A at Location 1 equal those in Type B at Location 2, and stocks in Type A at Location 2 equal those in Type B at Location 1 (Figure 3a). Because our meta‐ecosystem is spatially symmetrical, initial conditions can invert the pattern among locations (bistability), but the general source‐sink configuration stays the same (Figure 3c).

**FIGURE 4:**
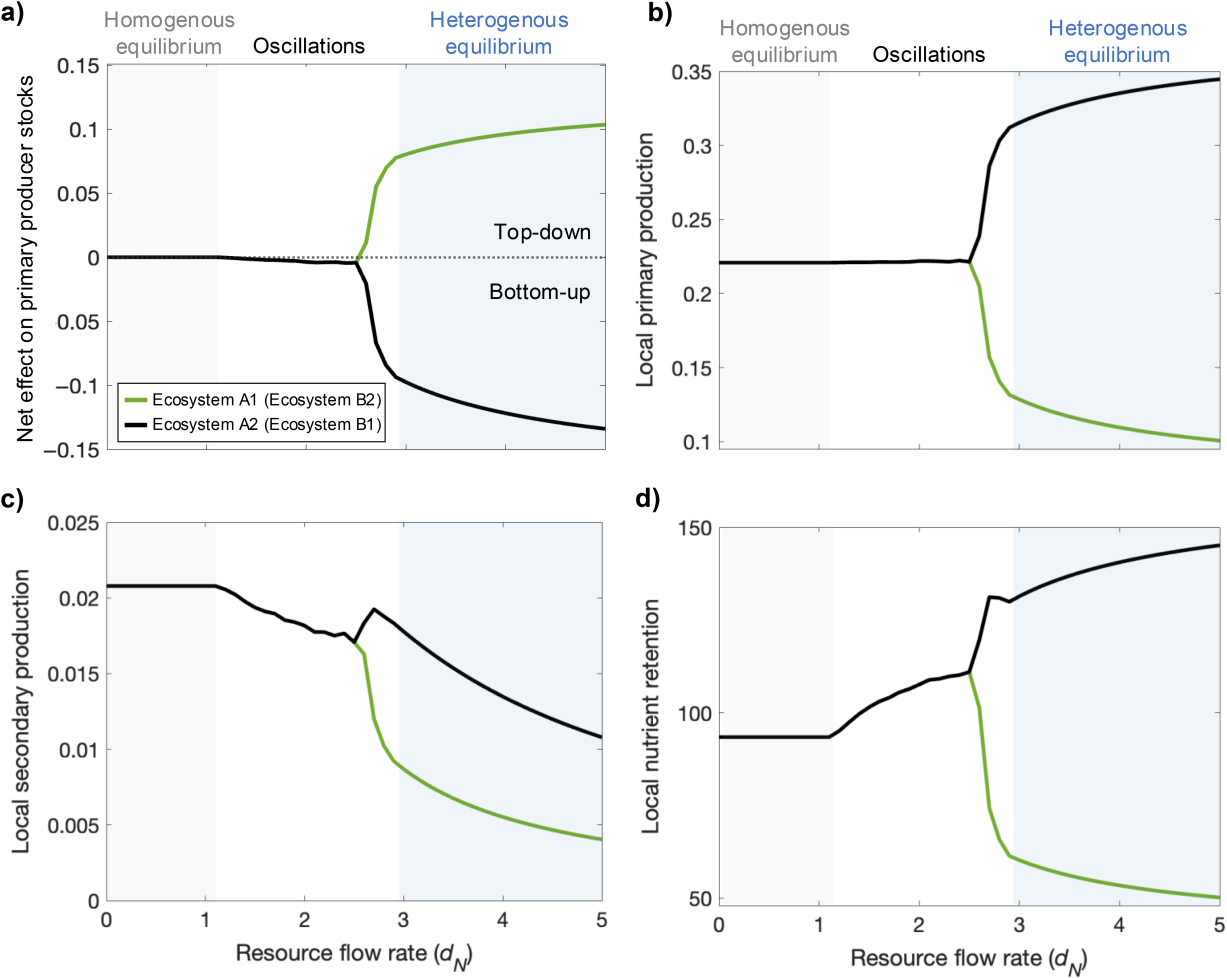
Local ecosystem (a) trophic regulation of primary producer stocks, (b) primary production, (c) secondary production, and (d) nutrient retention across increasing resource flow rates and stable states of the meta‐ecosystem. Spatial heterogeneity emerges at sufficiently high rates of resource flows (*d*_*N*_ ≥ 2.65) and dynamics are stabilized from slightly higher *d*_*N*_ (>2.95; blue background). In the spatially heterogeneous state, (a) one ecosystem of each type is subject to a net, top‐down impact (green line), while the other ecosystem of each type experiences a net, bottom‐up impact of spatial flows (black line). Local ecosystems subject to the top‐down impact have lower primary production (b; green line), secondary production (c; green line), and nutrient retention (d; green line), relative to the spatially homogeneous stable states (*d*_*N*_ < 2.65), whereas ecosystems subject to the bottom‐up impact have higher primary production (b; black line) and nutrient retention (d; black line), but generally lower secondary production (c; black line). In panel (a), the gray dashed line indicates the zero line where spatial flows have no net effect on trophic regulation. Parameter values used to generate the figure are *I*_*N*_ = 0.05, *e*_*N*_ = 0.01, *m*_*P*_ = 0.05, *m*_*C*_ = 0.24, *a*_*P*_ = 1, *b*_*P*_ = 10, *a*_*C*_ = 0.6, *b*_*C*_ = 6, *r*_*P*_ = 0.8, *r*_*C*_ = 0.8, and *d*_*C*_ = 0.4. Dispersal rates are equivalent for both ecosystem types. Observed impacts are identical across ecosystem types, thus a single line in the figure shows the result for one Type A and one Type B ecosystem (green lines: Type A Location 1 and Type B Location 2; black lines: Type A Location 2 and Type B Location 1).

The spatially heterogeneous equilibrium similarly exhibits spatially heterogeneous trophic regulation and ecosystem functions between ecosystems of the same type (Figure 4). Relative to the spatially homogeneous stable states (lower $d_{N}$), one ecosystem of each type has stronger top‐down regulation of primary producer stocks (Figure 4a, green line), whereas the other has stronger bottom‐up regulation (Figure 4a, black line). Local ecosystems with stronger top‐down regulation are those that emerge as sinks of consumers and sources of nutrients (Figure 3a) and have lower primary and secondary production (Figure 4b,c, green lines). Local ecosystems with stronger bottom‐up regulation are those that emerge as sources of consumers and sinks of nutrients (Figure 3a) and have higher primary production, but generally lower secondary production relative to the spatially homogeneous states at lower $d_{N}$ (Figure 4b,c, black lines). Local ecosystems with stronger bottom‐up regulation, however, have higher secondary production relative to local ecosystems with stronger top‐down regulation (Figure 4c, black vs. green line). Relative to the spatially homogeneous stable states, these differences in production, and the associated changes in primary producer stocks, result in greater total nutrient retention in local ecosystems that emerge as sources of consumers and lower total nutrient retention in ecosystems that emerge as sinks of consumers (Figure 4d). The observed impacts on local trophic regulation and ecosystem functions are identical for both ecosystem types.

### Local impacts of cross‐scale source‐sink dynamics scale up to drive meta‐ecosystem functions

**FIGURE 5:**
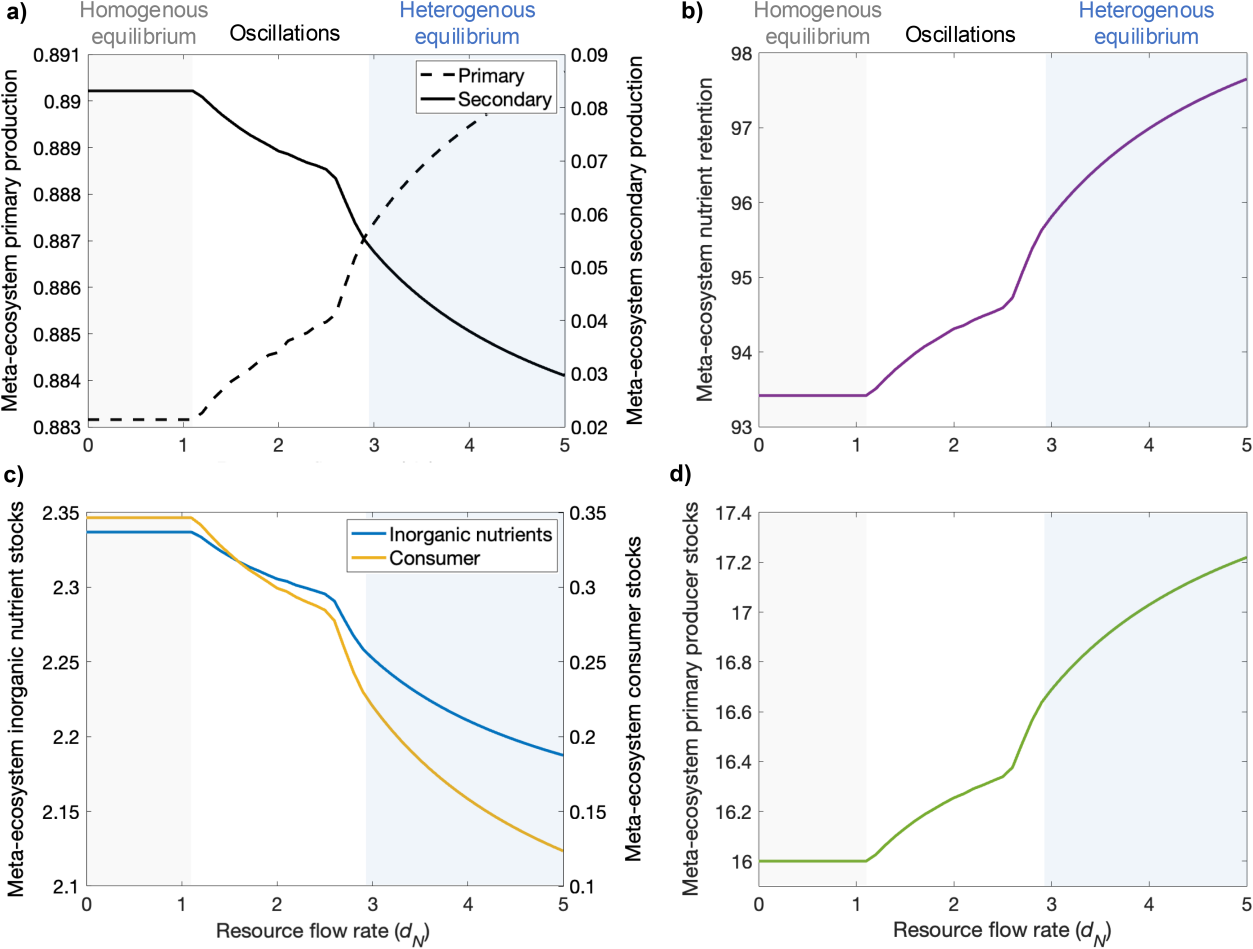
Meta‐ecosystem functions and stocks across increasing resource flow rates and stable states. (a) Meta‐ecosystem primary production increases (black dashed line) while secondary production decreases (black solid line) with increasing resource flow rates. (b) Meta‐ecosystem nutrient retention increases with resource flow rate. (c) Meta‐ecosystem inorganic nutrient and consumer stocks decrease, whereas (d) producer stocks increase relatively markedly with resource flow rates. Parameter values used to generate the figure are *I*_*N*_ = 0.05, *e*_*N*_ = 0.01, *m*_*P*_ = 0.05, *m*_*C*_ = 0.24, *a*_*P*_ = 1, *b*_*P*_ = 10, *a*_*C*_ = 0.6, *b*_*C*_ = 6, *r*_*P*_ = 0.8, *r*_*C*_ = 0.8, and *d*_*c*_ = 0.4. Dispersal rates are equivalent for both ecosystem types.

Cross‐scale source‐sink dynamics also influence ecosystem functions at the meta‐ecosystem level (Figure 5). Specifically, meta‐ecosystem primary production is higher at the spatially heterogeneous equilibrium relative to the spatially homogeneous equilibrium and oscillatory stable states (Figure 5a, dashed line), whereas meta‐ecosystem secondary production is lower (Figure 5a, solid line), resulting in higher primary producer but lower consumer and inorganic nutrient stocks (Figure 5c,d). The increase in primary production, however, is minimal compared to the decrease in secondary production. In addition, meta‐ecosystem nutrient retention is higher at the spatially heterogeneous equilibrium relative to the other stable states (Figure 5b).

### Ecosystem‐specific dispersal has cross‐ecosystem and meta‐ecosystem effects

**FIGURE 6:**
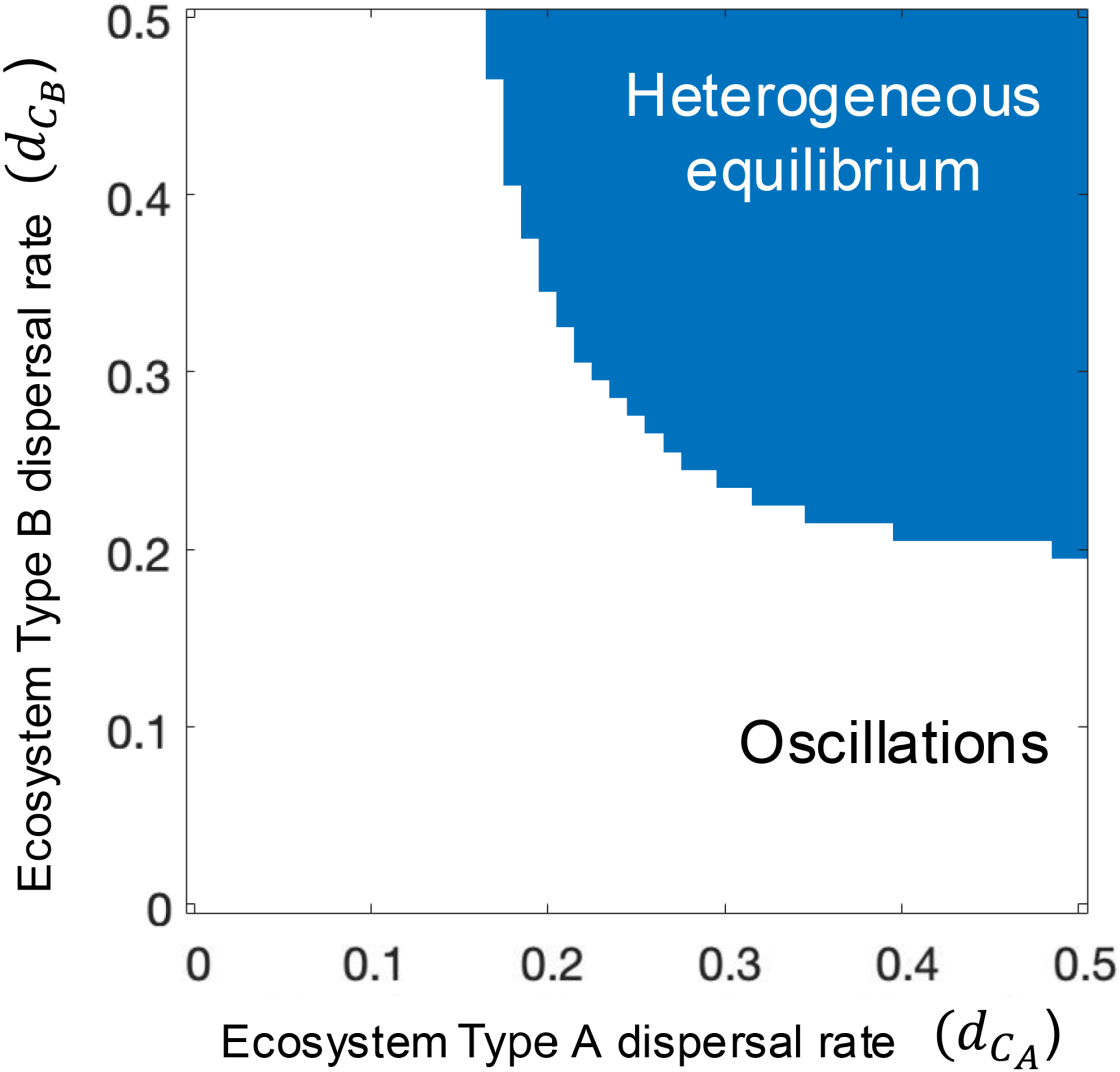
Ecosystem‐specific dispersal rates mediate the emergence of cross‐scale source‐sink dynamics and the spatially heterogeneous equilibrium in the meta‐ecosystem with the spatially nested topology of coupled ecosystems. The figure shows the stable states of the meta‐ecosystem for a single resource flow rate, *d*_*N*_ = 5, which is sufficiently high for cross‐scale source‐sink dynamics to emerge (see Figure 2). White areas indicate oscillatory dynamics and blue areas indicate a stable spatially heterogeneous equilibrium with cross‐scale source‐sink dynamics and ecosystem stocks differing across local ecosystems. Parameter values used to generate the figure are *I*_*N*_ = 0.05, *e*_*N*_ = 0.01, *m*_*P*_ = 0.05, *m*_*C*_ = 0.24, *a*_*P*_ = 1, *b*_*P*_ = 10, *a*_*C*_ = 0.6, *b*_*C*_ = 6, *r*_*P*_ = 0.8, *r*_*C*_ = 0.8, and *d*_*N*_ = 5.

Holding the resource flow rate constant (*d*_*N*_ = 5), we further show that the stable state of the meta‐ecosystem depends on dispersal rates of consumers in both ecosystem types (Figure 6). Specifically, the dispersal rate for both ecosystem types must be above a critical rate in order for cross‐scale source‐sink dynamics to emerge. Decreasing the consumer dispersal rate for either ecosystem type, there is a critical point below which source‐sink dynamics do not emerge and thus the spatially heterogeneous equilibrium is not reached. In Appendix S1: Section S8, we show that the rate of resource flows impacts the critical dispersal rates required for the emergence of source‐sink dynamics. At lower resource flow rates, dispersal rates of both ecosystem types need to be higher for source‐sink dynamics to emerge, relative to when resource flow rates are higher.

## DISCUSSION

Here, we used a meta‐ecosystem model to study the significance of the spatially nested topology of coupled ecosystems observed in nature, whereby non‐living resource flows couple different ecosystem types across smaller distances than consumer dispersal, which couples similar ecosystem types (Gounand et al., 2018). We showed that this spatially nested topology of coupled ecosystems allows for the emergence of cross‐scale source‐sink dynamics across ecosystem types, which has a general stabilizing effect on meta‐ecosystem dynamics. This stabilization yields a spatially heterogeneous stable equilibrium state that is characterized by spatial heterogeneity in local trophic regulation of primary producer stocks and ecosystem functions, which scale up to influence functions at the meta‐ecosystem scale. Research investigating the topology of spatial flows coupling ecosystems typically assumes a single type of spatial flow or that different types of spatial flows couple the same ecosystem patches (Gilarranz & Bascompte, 2012; Gounand et al., 2014; Peller et al., 2022). Ultimately, our study suggests that the distinguishing characteristics of the different types of spatial flows coupling ecosystems in nature are important properties that can drive the dynamics and functioning of ecosystems across scales.

Our study predicts that cross‐scale source‐sink dynamics can emerge from the contrasting nature and spatial scale of resource flows versus dispersal, to stabilize meta‐ecosystems. In the source‐sink structure that emerged, a single ecosystem is a sink (source) of inorganic nutrients for nearby ecosystems of different types while simultaneously being a source (sink) of consumers for relatively distant ecosystems of similar type. Traditionally, the concept of source‐sink dynamics was developed with a focus on dispersal between ecosystems of similar types, where ecosystems are described as sources or sinks based upon intrinsic rates of population growth (Pulliam, 1988). More recently, meta‐ecosystem models with two ecosystems coupled via both resource flows and dispersal have shown that ecosystems can simultaneously be sources and sinks of different ecosystem compartments (based on the net direction of spatial flows) (Gravel et al., 2010). In nature, however, different types of spatial flows often couple ecosystems across different spatial scales and different ecosystem types (Gounand et al., 2018). By integrating this common feature of nature into a meta‐ecosystem model, our study provides a novel perspective on source‐sink dynamics. Specifically, we show that source‐sink dynamics can operate across spatial scales and ecosystem types, yielding interdependencies between diverse sets of ecosystems, which have implications for the stability and functions of heterogeneous landscapes.

Our findings expand our understanding of a fundamental mechanism stabilizing ecosystem dynamics—the nutrient storage mechanism (Gounand et al., 2014)—demonstrating that it can operate in a distinct way in systems with different types of spatial flows. The nutrient storage mechanism was previously documented in meta‐ecosystems with two ecosystems coupled via dispersal. In these systems, strong top‐down control of primary producers in a consumer dispersal sink limits the uptake of nutrients by primary producers, allowing excess nutrients to be stored in inorganic form. This nutrient storage can stabilize meta‐ecosystems by limiting two drivers of destabilization: overproduction in top‐down controlled consumer sinks, and overcompensation in bottom‐up controlled consumer sources (Gounand et al., 2014). Here, we show that in multi‐scale meta‐ecosystems with different types of spatial flows, this mechanism can stabilize the system while allowing an increase in primary production (Figure 5a). The source‐sink dynamics we report are characterized by strong bottom‐up control in ecosystems that are sources of consumers and sinks of inorganic nutrients and that are connected to ecosystems with relatively strong top‐down control and the opposite source‐sink status of ecosystem compartments. This asymmetry in the strength of trophic control among connected ecosystems (Figure 4a) leads to the directional circulation of nutrients (in living and non‐living form) around the meta‐ecosystem (Figure 3a), which creates a spatial feedback loop that reinforces the spatially heterogeneous stable equilibrium. More specifically, excess consumers that would have destabilized the system spill over from more productive consumer sources to relatively unproductive consumer sinks, where nutrients are stored, decreasing local consumer production and total consumer biomass in the meta‐ecosystem (Figures 4c and 5c, respectively). However, the potential for inorganic nutrient accumulation is reduced due to nutrient flows connecting adjacent ecosystems of different types: excess inorganic nutrients in unproductive consumer sinks flow across ecosystem boundaries to more productive neighboring ecosystems. This spatial flow of inorganic nutrients slightly relaxes the nutrient storage effect while redistributing nutrients to more productive ecosystems within the meta‐ecosystem. Ultimately, these dynamics benefit primary producers but still prevent destabilization of the meta‐ecosystem via consumer spillover.

The cross‐scale source‐sink dynamics that emerged in the spatially nested topology of coupled ecosystems were similarly characterized by spatially heterogeneous ecosystem functions, which scaled up to influence meta‐ecosystem functions. Since inorganic nutrient sinks were bottom‐up regulated, primary production was higher in these ecosystems (relative to inorganic nutrient sources), which corresponded to higher secondary production and a net dispersal of consumers to ecosystems with lower primary producer stocks. This spatial mismatch between consumer immigration and primary producer stocks benefited primary production at the meta‐ecosystem level, relative to the spatially homogeneous stable states where cross‐scale source‐sink dynamics did not occur. However, this spatial mismatch reduced secondary production at the meta‐ecosystem level, as it led to a decrease in the consumption of primary producers by consumers across all local ecosystems. It is well established that resource flows can positively impact biomass and production in recipient ecosystems at local scales (Montagano et al., 2019; Polis et al., 1997). Our findings align with this, while also contributing to growing evidence that the emergent effects of resource flows at the meta‐ecosystem level may include trade‐offs or even reductions in certain functions (Harvey et al., 2023; Peller et al., 2024; Pichon et al., 2023).

Our study predicts that the emergence of cross‐scale source‐sink dynamics—and their functional implications—depends on the dispersal rates of *both* ecosystem types in the meta‐ecosystem, in addition to resource flow rates. While high resource flow rates are needed to generate the conditions for spatial heterogeneity, sufficiently high dispersal rates for both ecosystem types are required for adequate consumer spillover to stabilize the oscillations in source‐sink dynamics. Notably, when the meta‐ecosystem is oscillatory, but near the transition to the spatially heterogeneous equilibrium, increases in the dispersal rates of either ecosystem type *or* resource flows can drive the transition. This is because increasing resource flow rates increases production via stronger bottom‐up effects (Peller et al., 2024) and, in turn, enhances the magnitude of consumer dispersal. Dispersal has been widely integrated into ecological theory, including theory seeking to inform the design of protected area networks (e.g., Anadón et al., 2013; Melià et al., 2016). However, as dispersal generally couples ecosystems of similar types, such theory has largely overlooked the potential for dispersal to have cross‐ecosystem effects. By integrating dispersal and cross‐ecosystem resource flows into a meta‐ecosystem model, our findings show that dispersal processes specific to one ecosystem type can indirectly affect the stability and functions of different ecosystem types by affecting the dynamics of the meta‐ecosystem they are all part of. As cross‐ecosystem flows are ubiquitous in nature (Peller et al., 2024; Polis et al., 1997), our findings call for an improved understanding of their impacts in dispersal networks. Further, they suggest the need for a spatial ecosystem perspective for conservation strategies—one that accounts for multiple ecosystem types and the different types of spatial flows linking them.

### Links to empirical systems

Our results, although theoretical, find grounding in empirical systems. For instance, evidence from coral reefs supports the potential for the nutrient storage mechanism to stabilize ecosystems. In particular, Savage (2019) showed that on coral reefs with abundant herbivorous fish controlling macroalgal growth through top‐down regulation, nutrient flows from adjacent islands do not result in macroalgal overgrowth despite high observed local nutrient concentrations. As empirical investigations that simultaneously study resource flows and dispersal remain scarce, such observations have not been directly linked to dispersal. However, they do show the potential for dispersal, via its effect on trophic control, to influence how resource flows are used and stored. Although the potential export of stored nutrients was not studied in this example, in other systems, such as wetlands, research has shown that top‐down control can modulate not just nutrient storage but also nutrient export (Wyatt et al., 2021), reinforcing the idea that top‐down regulation—and by extension, dispersal—can shape the fate of nutrients at broader scales. Notably, meta‐analyses have demonstrated the context dependency of interactions between top‐down and bottom‐up forces, suggesting outcomes can vary across systems with differences in inherent productivity and across functional groups of primary producers (Burkepile & Hay, 2006; Hillebrand, 2002). Ultimately, this suggests the effect of the nutrient storage mechanism in real‐world meta‐ecosystems could vary across latitudes and ecosystem types.

Our findings can also help explain a widespread empirical pattern: spatial heterogeneity in ecosystem structure and function among ecosystems of the same type. Observational studies across diverse systems have long documented spatial heterogeneity in ecosystem structure and function, as well as trophic regulation (e.g., Burkepile & Hay, 2006; Gripenberg & Roslin, 2007; Turner & Chapin, 2005), which cannot be explained by local conditions alone. Increasingly, spatial flows of resources and organisms are, independently of each other, recognized as drivers of such heterogeneity. For example, algae and herbivore biomass in ponds with similar characteristics can vary across landscapes, which has been linked to differences in dispersal connectivity (Chase et al., 2010). Similarly, coral reefs located in comparable oceanographic settings can exhibit variation in fish biomass and primary production that has been linked to spatial variation in resource subsidies from adjacent terrestrial ecosystems (Benkwitt et al., 2021; Graham et al., 2018). While both resource flows and dispersal are increasingly recognized as drivers of spatial heterogeneity, few studies have considered how their combined influence shapes ecosystem structure and function. Our study provides a mechanistic explanation for how the interaction of resource flows and dispersal—operating across different spatial scales and ecosystem types—can generate the spatial heterogeneity in ecosystem structure and function ubiquitously observed in nature. In doing so, it reveals how landscape‐level patterns can emerge from the interplay of processes often studied in isolation.

While our study focused on the spatially nested topology, where resource flows link ecosystems at smaller spatial scales than consumer dispersal, there is empirical evidence to suggest that other spatial configurations occur in nature. For instance, in some systems, large‐scale resource flows, such as those driving coastal eutrophication in the Gulf of Mexico and the Baltic Sea (Bonsdorff et al., 1997) or floating *Sargassum* mats in the Atlantic (Wang et al., 2019), can operate over broader spatial scales than local organism dispersal, effectively inverting the nested structure we modeled. Other configurations, such as both resource flows and dispersal occurring at similar scales or dispersal being more spatially localized, may give rise to distinct meta‐ecosystem dynamics with different ecological implications. Additionally, our analysis was limited to passive resource flows and consumer dispersal, but other forms of movement—such as ontogenetic migrations—can result in large‐scale organism movement and nutrient transfer between habitats used at different life stages (e.g., Brandt et al., 2024; Jonsson & Jonsson, 2003; Peller et al., 2023). Incorporating such forms of movement, as well as different spatial topologies, into meta‐ecosystem models may reveal a rich set of dynamics and further improve our understanding of ecosystem functioning across complex landscapes.

Our theoretical predictions could be tested using experimental meta‐ecosystems, which have been increasingly used to test predictions on dispersal and resource flow effects (e.g., Giacomuzzo et al., 2024; Gülzow et al., 2019; Harvey et al., 2020). Building on commonly used two‐patch designs (Giacomuzzo et al., 2024; Gounand et al., 2017), aquatic microcosm or mesocosm systems composed of jars or tanks could be extended to a four‐patch design reflecting the spatially nested topology analyzed here. As an example, individual microcosms could contain simple food chains, such as *Daphnia magna* as a consumer and *Euglena gracilis* as a primary producer (Gounand et al., 2017). Resource flows could be imposed via the transfer of nonliving material between microcosms (e.g., boiled or microwaved volume), while consumer dispersal could be imposed via the manual transfer of consumers. By independently varying the rates of these transfers, experiments could test whether strong contrasts between the rates of resource flows and dispersal lead to stabilized dynamics and spatial heterogeneity in ecosystem biomass structure.

### Concluding remarks

To date, little progress has been made toward a mechanistic understanding of ecosystem functioning integrating diverse ecosystem types and the topology of their interactions via spatial flows of living and nonliving ecosystem compartments. By studying commonly observed constraints on the topology of interactions among diverse, spatially coupled ecosystems, we have shown that the meta‐ecosystem perspective provides a powerful framework to develop this understanding. Future research in this direction will reveal how the diversity of ecosystems and the structure of spatial flows between them jointly shape ecosystem functioning—insights that are crucial for managing ecosystems in a changing world.

## AUTHOR CONTRIBUTIONS

All authors conceptualized the study. Tianna Peller carried out all analyses and wrote the first manuscript draft. All authors contributed substantially to revising the manuscript.

## CONFLICT OF INTEREST STATEMENT

The authors declare no conflicts of interest.

## Supporting information


Appendix S1.

## Data Availability

Code (Peller, 2024) is available in Zenodo at https://doi.org/10.5281/zenodo.12529602.
