# Supplementary material for: Spatially‐nested topologies stabilize meta‐ecosystems via cross‐scale source‐sink dynamics
Source: Ecology. 2026 Apr 12;107(4):e70375. doi: 10.1002/ecy.70375 (PMC13071351; doi:10.1002/ecy.70375)
Supplement: Supplementary file 1 — Appendix S1. [file ECY-107-e70375-s001.pdf]

Appendix S1 for: Spatially-nested topologies stabilize meta-ecosystems via cross-scale source-sink dynamics

Authors: Tianna Peller, Isabelle Gounand, Marie-Josée Fortin, Frédéric Guichard

Ecology

Table of Contents

Section S1: Definitions and units of state variables and parameters 3

Section S2: Stable states of the meta-ecosystem when primary producer dispersal > 0 4

Section S3: Expanded range of initial conditions 6

Section S4: Characterizing the other stable states in the meta-ecosystem..................................... 9

Section S5: Extrema of regional primary producer and consumer stocks across an expanded range of resource flow rates 13

Section S6: Spatially-nested topologies of coupled ecosystems for meta-ecosystems of different size and shape 15

Section S7: Non-equilibrium source-sink dynamics and temporally fluctuating net resource flows 18

Section S8: Ecosystem-specific dispersal rates 20

Section S9: Consumer dispersal effects 21

Section S1: Definitions and units of state variables and parameters

Table S1. State variable and parameter table for the meta-ecosystem model.

State variable or parameter	Definition	Unit	Default value
N_i	Nutrient stock in inorganic form in ecosystem *i*	Quantity of nutrient	N/A
P_i	Nutrient stock in primary producers in ecosystem *i*	Quantity of nutrient	N/A
C_i	Nutrient stock in consumers in ecosystem *i*	Quantity of nutrient	N/A
I_N	Constant input to inorganic nutrient compartment	Time^{-1}·quantity of nutrient	0.05
e_N	Loss rate from inorganic nutrient compartment	Time^{-1}	0.01
m_P	Mortality rate of primary producers	Time^{-1}	0.05
m_C	Mortality rate of consumers	Time^{-1}	0.24
r_P	Proportion of nutrients from primary producer losses that are recycled locally	Unitless	0.8
r_C	Proportion of nutrients from consumer losses that are recycled locally	Unitless	0.8
d_N	Resource flow rate	Time^{-1}	0.0-5.0
d_C	Consumer dispersal rate	Time^{-1}	0.0-0.5
a_P	Primary producer maximum uptake rate	Time^{-1}	1.0
a_C	Consumer maximum attack rate	Time^{-1}	0.6
b_P	Primary producer half-saturation constant	Quantity of nutrient	10.0
b_C	Consumer handling time	Quantity of nutrient	6.0

Section S2: Stable states of the meta-ecosystem when primary producer dispersal > 0

In the main text, we assume primary producer dispersal occurs at the within ecosystem scale and that primary producers do not disperse between ecosystems at distant locations. This assumption is rooted in empirical evidence, which suggests that many primary producer species frequently disperse across relatively short distances (e.g., Kinlan & Gaines 2003, Nathan 2006, Schurr et al. 2009, Hosokawa et al. 2015). Infrequent long-distance dispersal events, however, have been documented in some primary producer species, suggesting primary producer dispersal has the potential to connect distant ecosystems through very low dispersal rates (Nathan 2006, Schurr et al. 2009). Thus, here we examine the effects of primary producers dispersing between the same ecosystems as consumers by setting primary producer dispersal rates greater than zero ($d_P > 0$). To do so, we modify the differential equation for primary producers used in the main text (equation 2) to include primary producer dispersal:

$$\frac{dP_i}{dt} = P_i f_{P_i}(N_i) - m_P P_i - C_i f_{C_i}(P_i) + d_P \sum_{j=1}^{n} s_{Pij} P_j$$

We follow the same procedure to identify stable states of the meta-ecosystem with the spatially-nested topology of coupled ecosystems that we described in the main text, but now with primary producers dispersing between the same ecosystems as consumers. At low rates of primary producer dispersal ($d_P \leq 0.0001$), we find it has little effect on the model dynamics: we observe a spatially-homogeneous equilibrium at low rates of resource flows, oscillatory dynamics with increasing rates of resource flows, and a spatially-heterogeneous equilibrium at high rates of both consumer dispersal and resource flows (Fig. S1a). Thus, in the context of the widespread observation that long-distance dispersal events by primary producers are infrequent, these predictions suggest the mechanisms and observations we describe in the main text are robust to

primary producer dispersal. Increasing primary producer dispersal rates, we find there is a critical point at which primary producer dispersal can influence the qualitative states observed. Specifically, for primary producer dispersal rates $d_P > 0.0001$, we do not observe the emergence of the spatially-heterogeneous equilibrium (Fig. S1b) because primary producer dispersal prevents the mechanism of stability.

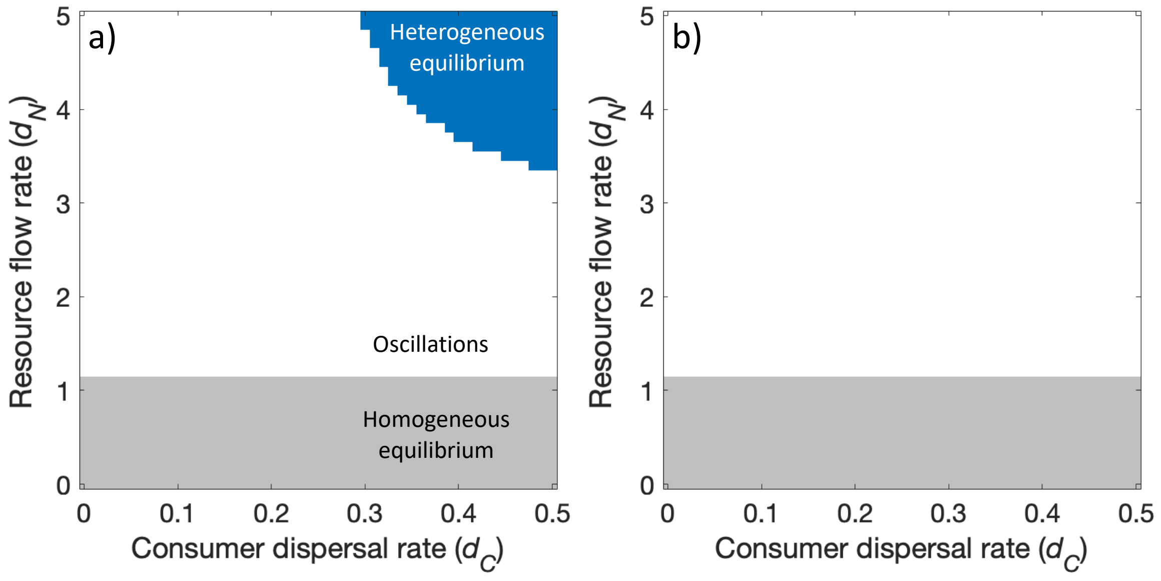

Figure S1. Stable states of the meta-ecosystem with primary producer dispersal ($d_P > 0$), across rates of consumer dispersal d_C and resource flows d_N. Primary producers disperse between ecosystems at rate (a) $d_P = 0.00005$ (b) $d_P = 0.00015$. Grey areas indicate a stable homogeneous equilibrium, where ecosystem stocks are identical across local ecosystems; white areas indicate oscillatory dynamics; and blue areas indicate a stable, spatially-heterogeneous equilibrium, where ecosystem stocks differ across local ecosystems. Parameters used to generate the figure are given in Figure 2 of the main text.

Section S3: Expanded range of initial conditions

For each combination of dispersal and resource flows rates we analyzed in the main text, we had randomly selected 50 initial conditions from a sequence between 0 and 10, which was the biologically feasible range for our system, based on the range of stock values we expected with the parameters used. To understand the behaviour of the model even beyond this biologically feasible range, here we analyze the model with an expanded range of initial conditions, selecting initial condition values up to an order of magnitude greater: from 0 to 100. We follow the same procedure to identify stable states of the meta-ecosystem with the spatially-nested topology of coupled ecosystems that we described in the main text. We show that expanding the range of initial conditions to include values that are substantially higher relative to the stocks reached in the model with the parameter sets used, reveals more than one alternative stable state at high rates of consumer dispersal and resource flows (purple zone, Fig. S2a). Specifically, in addition to the spatially-heterogeneous equilibrium described in the main text, we find a spatially-homogeneous equilibrium (Fig. S2b) and an unfeasible equilibrium (Fig. S2c). The spatially-homogeneous equilibrium has consumer stocks that are over an order of magnitude less than the other feasible equilibria observed (~0.001; Fig. S2b). At the unfeasible equilibrium, consumers cannot persist in the meta-ecosystem; only primary producers and inorganic nutrients attain positive stock values (Fig. S2c).

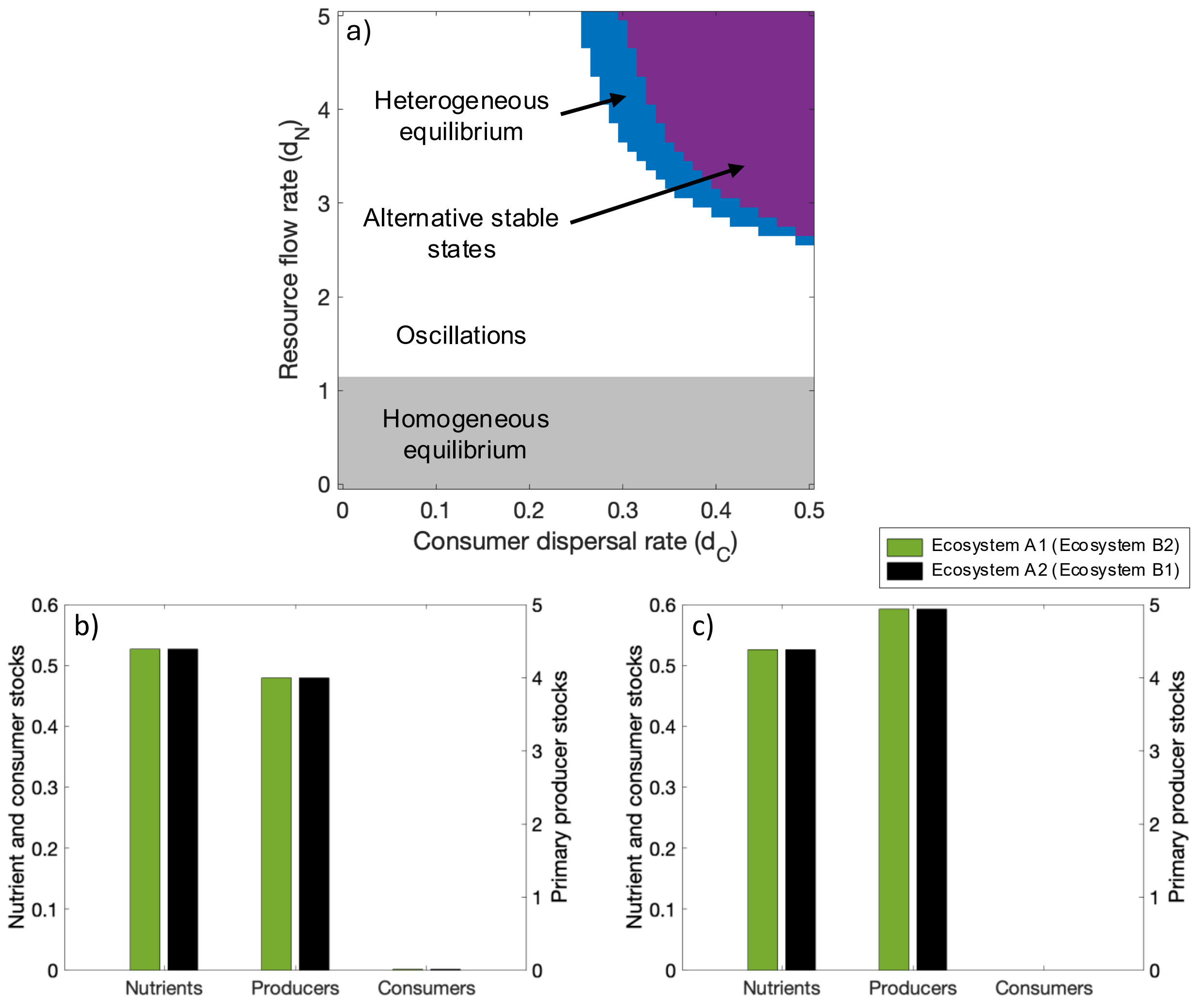

Figure S2. (a) Stable states of the meta-ecosystem across rates of consumer dispersal d_C and resource flows d_N, for an extended range of initial conditions, from 0 to 100. Grey, white, and blue areas correspond to the stable states that we described in the main text: grey areas indicate a stable homogeneous equilibrium, where ecosystem stocks are identical across local ecosystems; white areas indicate oscillatory dynamics; and blue areas indicate a stable heterogeneous equilibrium, where ecosystem stocks differ across local ecosystems. Purple areas indicate multiple alternative stable states: the spatially-heteregeneous equilibrium, and a spatially-homogeneous equilibrium and unfeasible equilibrium not observed for the range of initial conditions considered in the main text. Local ecosystem stocks for all ecosystems in the meta-

ecosystem at the (b) spatially-homogeneous equilibrium (purple zone in panel a) and (c) unfeasible equilibrium (purple zone in panel a) that can be reached for the extended range of initial conditions. The pattern of local ecosystem stocks is the same for the different ecosystem types, thus, each bar represents the stocks for an ecosystem compartment of one Type A and one Type B ecosystem (green bars: Type A location 1 (A1) and Type B location 2 (B2); black bars: Type A location 2 (A2) and Type B location 1 (B1)). Parameter values used to generate all panels are given in Figure 2 in the main text, except $d_C = 0.5$ and $d_N = 5$ in panels (b) and (c).

Section S4: Characterizing the other stable states in the meta-ecosystem

In the main text, our primary focus is the spatially-heterogeneous equilibrium, given it is unique to the spatially-nested topology of coupled ecosystems. Here, we show the dynamics of the other stable states of the spatially-nested meta-ecosystem described in Fig. 2a of the main text: the spatially-homogeneous equilibrium (Fig. S3), and the oscillatory stable states (Fig. S4), which can be either spatially-homogeneous, where ecosystems of the same type have identical stock values (Fig. S4a) or spatially-heterogeneous, where ecosystems of the same type differ in their stock values (Fig. S4b). For clarity, we illustrate the spatially-homogeneous equilibrium using a box plot showing stock values at equilibrium and the oscillatory stable states using time series figures.

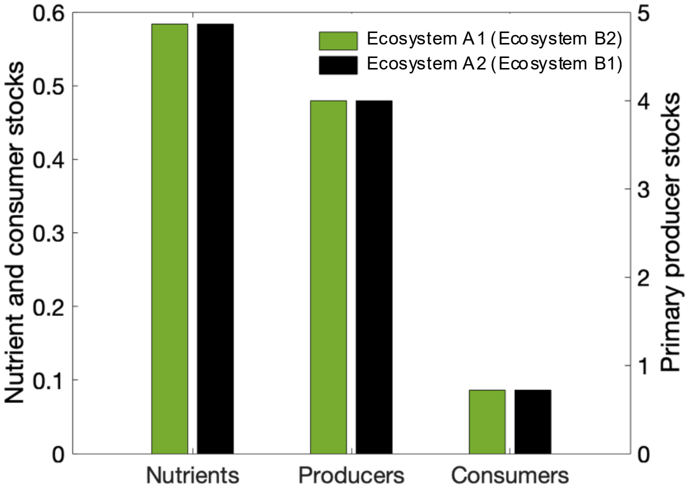

Figure S3. Local ecosystem stocks in the spatially-nested meta-ecosystem at the spatially-homogeneous equilibrium illustrated in Fig. 2a of the main text (grey zone) for resource flow rates $d_N < 1.1$. The pattern of local ecosystem stocks is the same for the different ecosystem types, thus, each bar represents the stocks for an ecosystem compartment of one Type A and one Type B ecosystem (green bars: Type A location 1 (A1) and Type B location 2 (B2); black bars: Type A location 2 (A2) and Type B location 1 (B1)). Parameters used to generate the figure are given in Figure 2 of the main text, except $d_C = 0.4$ and $d_N = 1.0$.

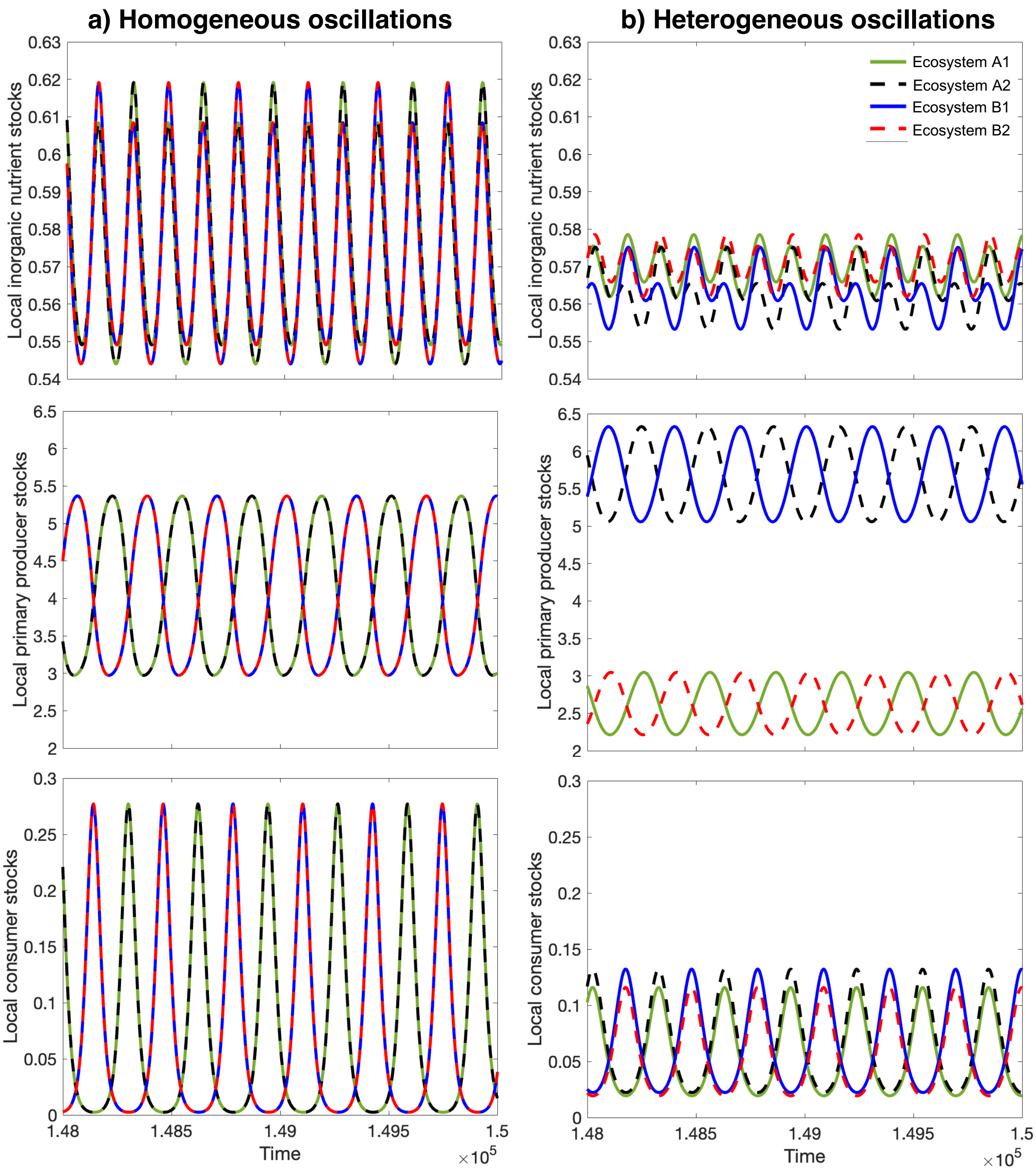

Figure S4. Local ecosystem stocks in the spatially-nested meta-ecosystem at the oscillatory states illustrated in Fig. 2a of the main text (white zone). Oscillations can be (a) spatially-homogeneous, where ecosystems of the same type (e.g., Ecosystem Type A location 1 (A1) and location 2 (A2)) have identical stock values or (b) spatially-heterogeneous, where ecosystems of

the same type differ in their stock values. Spatially-heterogeneous oscillations occur for restricted resource flow rates in close proximity to rates yielding the spatially-heterogeneous equilibrium. Parameters used to generate the figure are the same as Fig. S3, except in (a) $d_N = 2.4$ and in (b) $d_N = 2.8$.

Section S5: Extrema of regional primary producer and consumer stocks across an expanded range of resource flow rates

Here we show the consumer extrema across an expanded range of resource flow rates, as well as the corresponding figure for primary producer stocks (Fig. S5). Figure S5a shows the consumer minimum and maximum stock values at the meta-ecosystem level (i.e., consumer stocks summed across all four local ecosystems), and demonstrates that, across resource flow rates, consumer minimum values in the meta-ecosystem are generally higher at the spatially-heterogeneous equilibrium than they are at the oscillatory stable state. Similarly, Figure S5c shows that primary producer minimum values are higher at the spatially-heterogeneous equilibrium than they are for both the spatially-homogeneous equilibrium and oscillatory states. Figure S5b and S5d show that the meta-ecosystem with the fully-connected resource flow topology does not reach the spatially-heterogeneous equilibrium state across the expanded range of resource flow rates. Figure S5 shows resource flow rates up to a maximum of $d_N = 40$ to allow minimum stock values to be readily observed (higher resource flow rates lead to higher maxima for the oscillatory state, which expands the y-axis, making minima more difficult to observe). However, we note that we examined resource flow rates up to $d_N = 100$, across which the meta-ecosystem with the fully-connected resource flow topology does not reach the spatially-heterogeneous equilibrium.

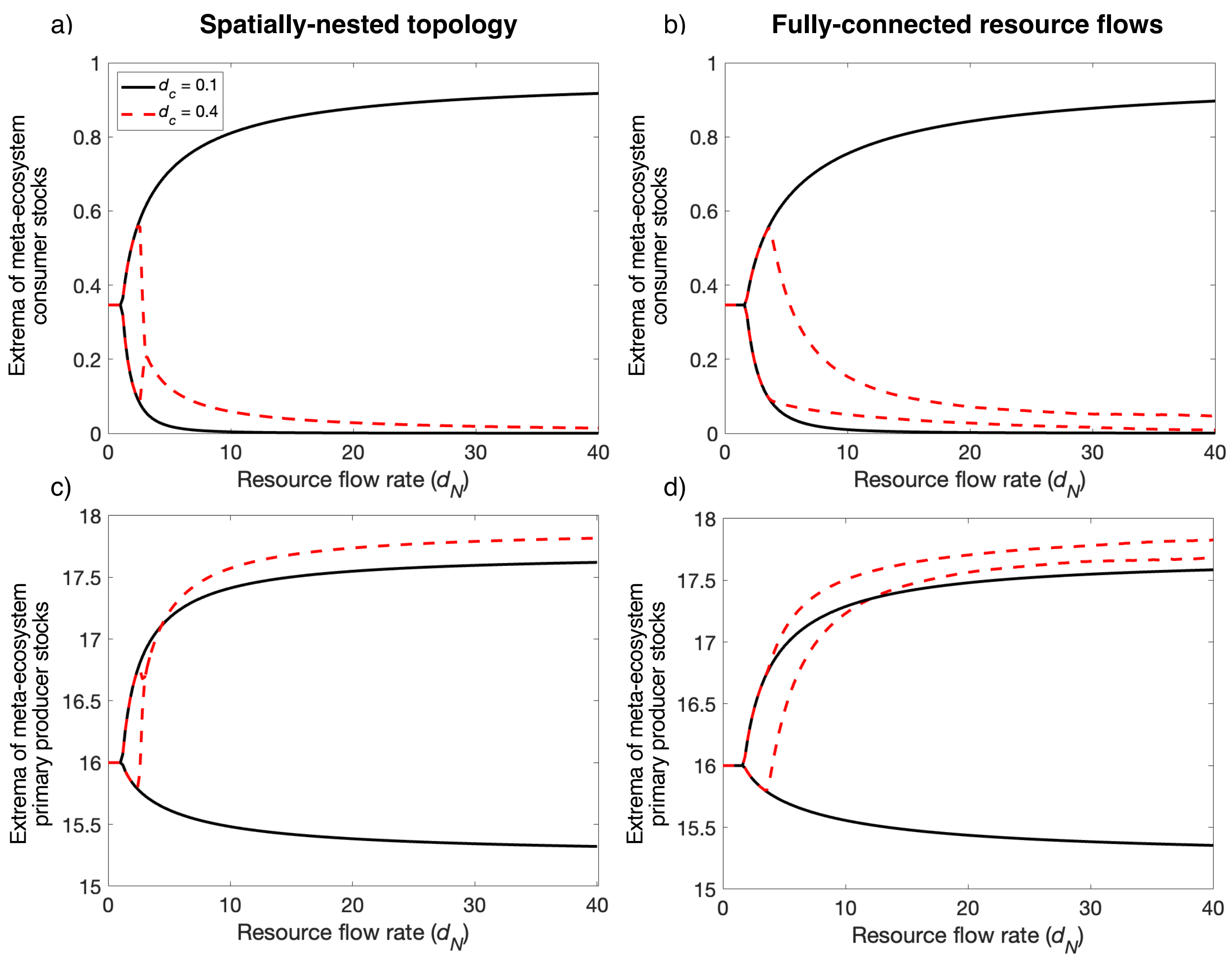

Figure S5. Bifurcation diagram of meta-ecosystem level consumer (a,b) and primary producer (c,d) minimum and maximum stocks values in the meta-ecosystem with the spatially-nested topology of coupled ecosystems (a,c) and fully-connected resource flows (b,d), across an expanded range of resource flow rates d_N. Extrema are shown for dispersal rates $d_C = 0.1$ (black lines) and $d_C = 0.4$ (red dashed lines), with the dispersal rate equivalent for both ecosystem types. Parameters used to generate the figure are given in Figure 2 of the main text.

Section S6: Spatially-nested topologies of coupled ecosystems for meta-ecosystems of different size and shape

To investigate the generality of our findings, here we expand our analysis to meta-ecosystems of larger size (eight ecosystems; Fig. S6a, Fig. S7a,b) and a looped rather than linear spatial structure (with six ecosystems; Fig. S6b, Fig. S7c,d). Figure S7 shows consistent predictions with the four-ecosystem spatial topologies of coupled ecosystems studied in the main text. Specifically, spatially-nested structures of coupled ecosystems, where resource flows couple ecosystems across smaller distances than dispersal, allow the emergence of a spatially-heterogeneous equilibrium state in both the linear eight-ecosystem meta-ecosystem (Fig. S7a) and looped six-ecosystem meta-ecosystem (Fig. S7c). Whereas, when these meta-ecosystems instead have fully-connected resource flows, where each ecosystem in the meta-ecosystem is connected to all other ecosystems via resource flows, this spatially-heterogeneous equilibrium does not emerge (Fig. S7b,d).

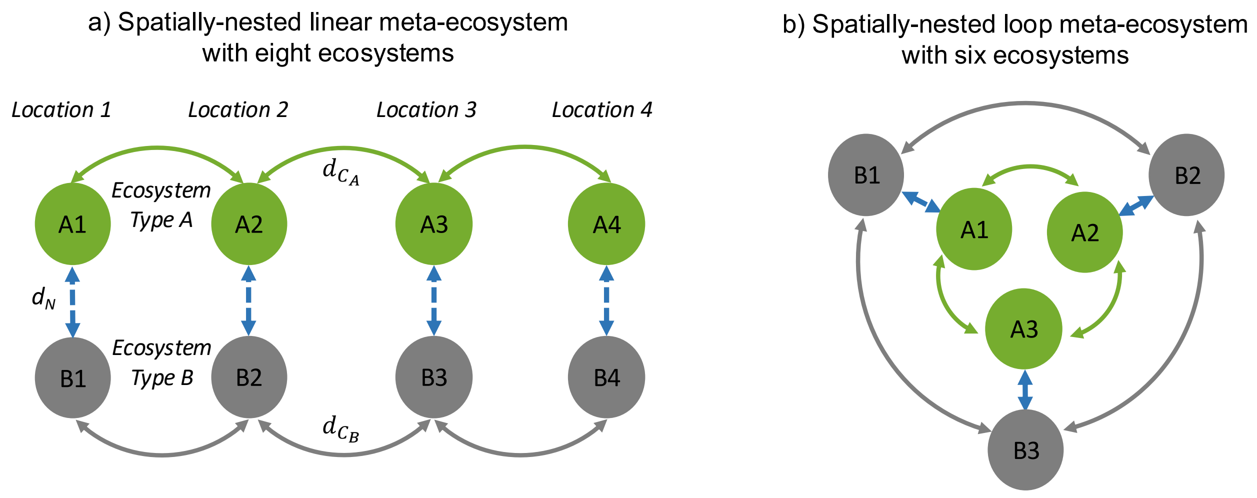

Figure S6. Conceptual diagram of meta-ecosystem models with (a) eight ecosystems in a linear spatially-nested topology of coupled ecosystems and (b) six ecosystems in a looped spatially-nested topology of coupled ecosystems. As in the main text, the meta-ecosystems are general, but we present the results for the concrete example shown consisting of two ecosystem types: Type A (green) and Type B (grey), each exhibiting ecosystem-specific dispersal rates d_{C_A} and d_{C_B}, respectively. In both (a) and (b), dispersal connects ecosystems of the same type at different locations across larger distances relative to resource flows, which connect nearby ecosystems of different types. Control meta-ecosystems with fully-connected resource flows — all ecosystems are connected by resource flows, regardless of distance — were also studied for each spatial topology, but not shown here due to the lack of clarity resulting from the many required and overlapping resource flow arrows.

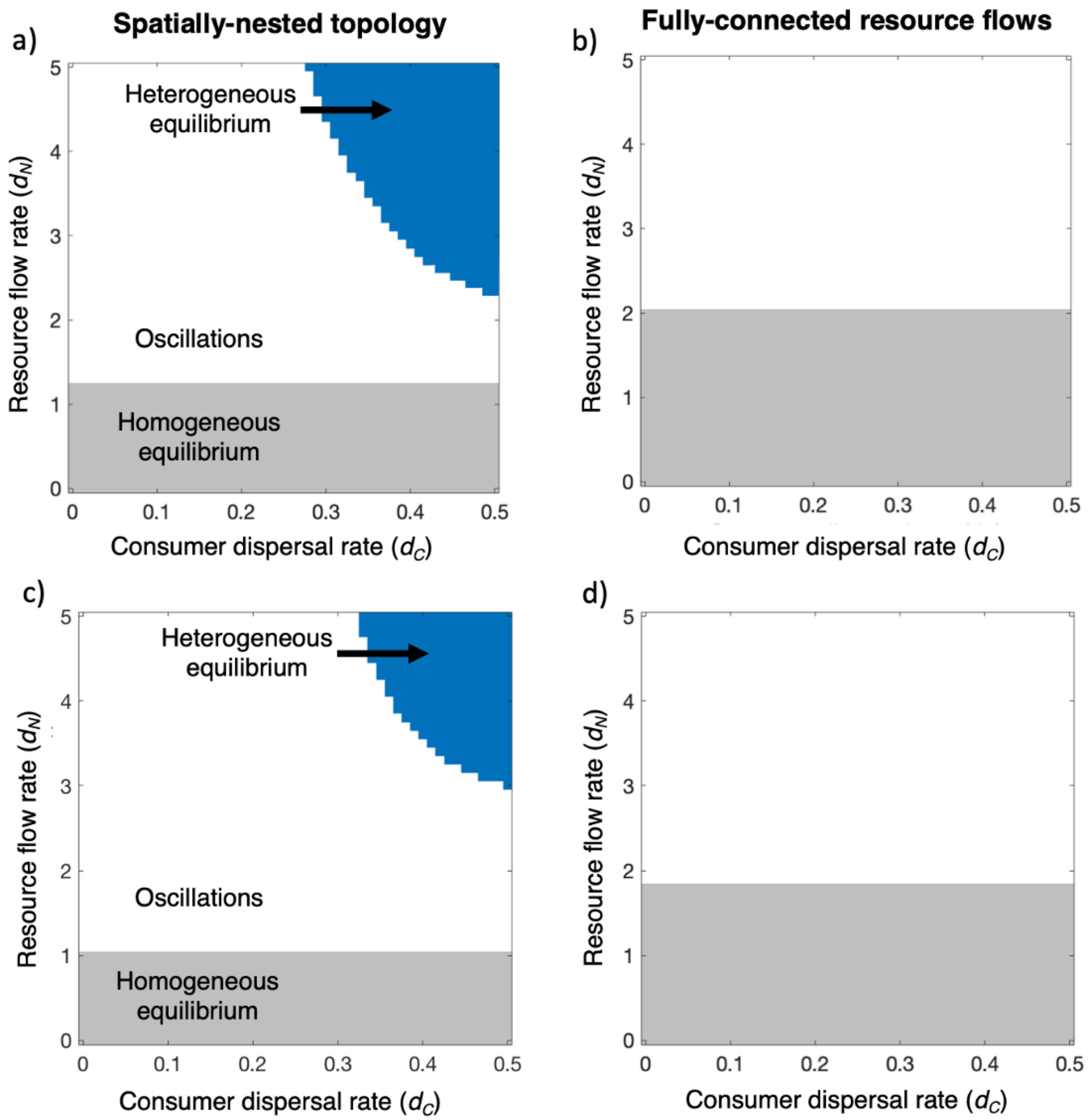

Figure S7. Qualitative states across rates of consumer dispersal d_C and resource flows d_N, for the linear eight-ecosystem meta-ecosystem (a,b; see Fig. S6a for spatial structure) and the looped six-ecosystem meta-ecosystem (c,d; see Fig. S6b for spatial structure) with a spatially-nested topology of coupled ecosystems (a,c) and fully-connected resource flows (b,d). Grey areas indicate a stable homogeneous equilibrium, where ecosystem stocks are identical across local ecosystems; white areas indicate oscillatory dynamics; and blue areas indicate a stable heterogeneous equilibrium, where ecosystem stocks differ across local ecosystems.

Section S7: Non-equilibrium source-sink dynamics and temporally fluctuating net resource flows

We indicated in the main text that the equilibrium cross-scale source-sink dynamics that are observed in the meta-ecosystem with the spatially-nested topology of connected ecosystems are not reached in the meta-ecosystem with fully-connected resource flows. Equilibrium source-sink dynamics are not reached in the meta-ecosystem with fully-connected resource flows because each ecosystem has a resource flow connection where the direction of the net flow fluctuates across time (Fig. S8a), preventing equilibrium source-sink dynamics. This leads to non-equilibrium source-sink dynamics in meta-ecosystems with fully-connected resource flows, where the ecosystem that is a source/sink of resources fluctuates across time.

In Figure S8b, we show how ecosystems with temporally fluctuating direction of net resource flows in the meta-ecosystem with fully-connected resource flows are characterized by positive covariance between resource flows and primary producer growth when the meta-ecosystem is at the oscillatory state (red dashed line, $d_N > 1.6$). We show that this positive covariance is also observed in the meta-ecosystem with the spatially-nested topology of coupled ecosystems (black line) when it is at its oscillatory state ($d_N = 1.2 - 2.8$), but that this positive covariance is lost when it transitions to the heterogeneous equilibrium ($d_N > 2.8$). Marleau et al. (2010) previously described how positive covariance between resource flows and primary producer growth results in the loss of a stable equilibrium: when primary producer growth is higher, there can be a net flow of nutrients into an ecosystem (because primary producer growth depletes nutrients), which feeds back to positively impact primary producer growth. Our results suggest this positive covariance between resource flows and primary producer growth in the meta-ecosystem with fully-connected resource flows hinders the stabilization of the oscillations that we observe in the meta-ecosystem with the spatially-nested topology of coupled ecosystems.

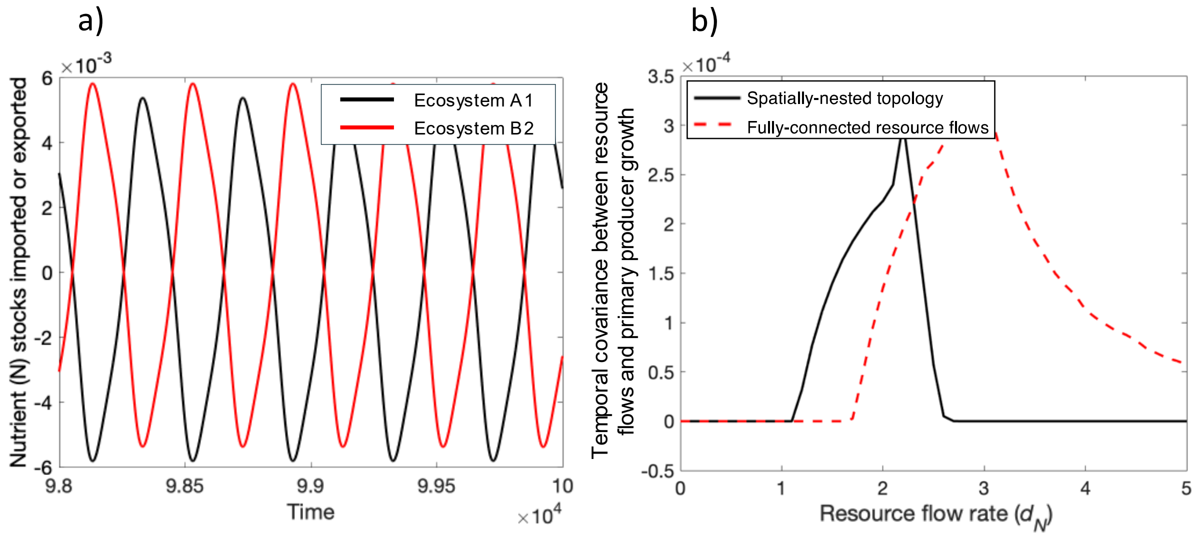

Figure S8. (a) In the meta-ecosystem with fully-connected resource flows, ecosystem Type A location 1 (A1; black line) and Type B location 2 (B2; red line) are connected through resource flows with a temporally fluctuating direction: each ecosystem repeatedly transitions from being a net exporter (line below zero) to a net importer (line above zero) of inorganic nutrients to/from the other. (b) Covariance between resource flows and primary producer growth of ecosystems in the meta-ecosystem with the spatially-nested topology of coupled ecosystems (black line) and fully-connected resource flows (red dashed line). With an increasing rate of resource flows, the positive covariance is lost in the meta-ecosystem with the spatially-nested topology, but not in the meta-ecosystem with fully-connected resource flows. Parameters used to generate the figure are given in Figure 2 of the main text, except $d_C = 0.5$.

Section S8: Ecosystem-specific dispersal rates

In the main text, we show how the emergence of cross-scale source-sink dynamics depends on the dispersal rate of both ecosystem types. In Fig. S9, we further show the critical, ecosystem-specific dispersal rates required for the emergence of source-sink dynamics depends on the resource flow rate. At lower resource flow rates (Fig. S9a), dispersal rates of both ecosystem types need to be higher for source-sink dynamics to emerge, relative to when resource flows rates are higher (Fig. S9b).

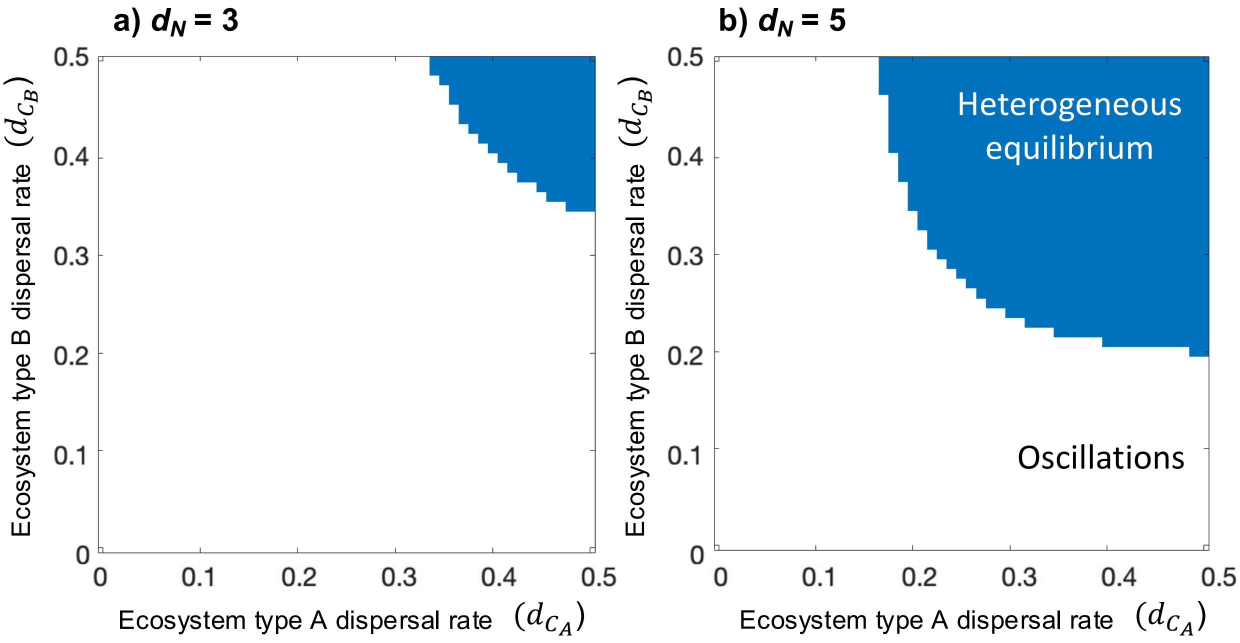

Figure S9. The emergence of cross-scale source-sink dynamics and the spatially-heterogeneous equilibrium depends on ecosystem-specific dispersal rates. The critical dispersal rate, of either ecosystem type, required to allow source-sink dynamics is higher when resource flow rates are lower (a) versus (b) higher. White areas indicate oscillatory dynamics and blue areas indicate a stable, spatially-heterogeneous equilibrium with cross-scale source-sink dynamics and ecosystem stocks differing across local ecosystems. Parameter values used to generate the figure are given in Figure 6 of the main text.

Section S9: Consumer dispersal effects

To better illustrate the effect of consumer dispersal on the meta-ecosystem's dynamics, we present the dominant eigenvalues of the equilibria (homogeneous and heterogeneous) across consumer dispersal rates in Figure S10. Figure S10 shows how increasing values of consumer dispersal can lead to positive eigenvalues becoming negative for the heterogeneous equilibrium (dashed lines) and highlights the dependency of this effect on the resource flow rate connecting ecosystems. Further, Figure S9 shows the dependence of local stability on consumer dispersal rates for the heterogeneous equilibrium (dashed lines) and its independence from consumer dispersal for the homogeneous equilibrium (solid lines).

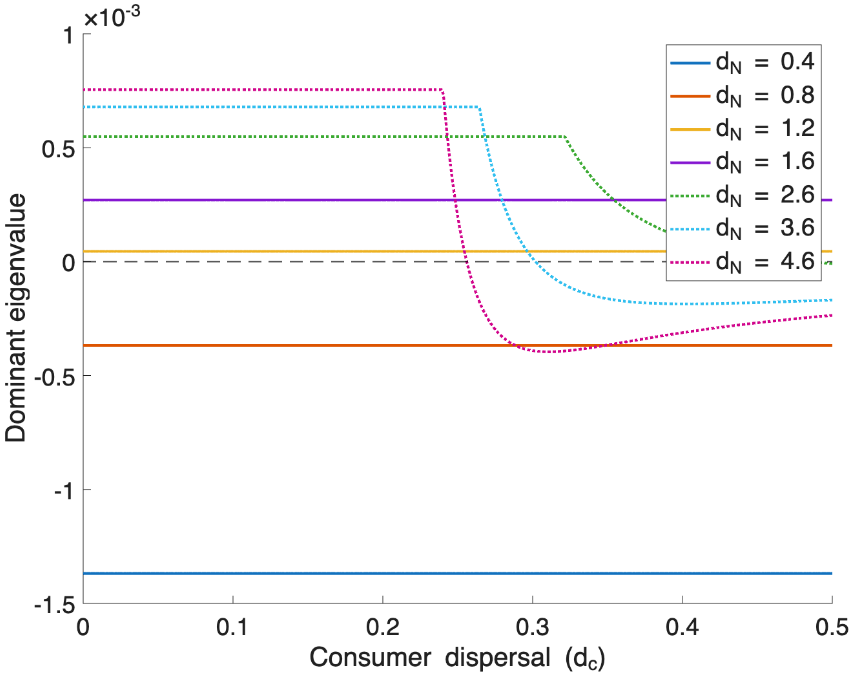

Figure S10. Dominant eigenvalue of the Jacobian evaluated at equilibrium as a function of consumer dispersal rate (d_C) for multiple fixed resource flow rates (d_N). Solid lines correspond to the spatially homogeneous equilibrium, while dashed lines correspond to the spatially heterogeneous equilibrium. Colors distinguish different values of d_N. The horizontal dashed line

at zero indicates the stability threshold: equilibria are locally stable where the dominant eigenvalue is negative and unstable where it is positive.

Literature Cited

Abbott, K.C. (2011). A dispersal-induced paradox: synchrony and stability in stochastic metapopulations. *Ecology Letters*, 14, 1158-1169.

Hosokawa, S., Nakaoka, M., Miyoshi, E., & Kuwae, T. (2015). Seed dispersal in the seagrass *Zostera marina* is mostly within the parent bed in a protected bay. *Marine Ecology Progress Series,* 523, 41-56.

Kinlan, B.P. & Gaines, S.D. (2003). Propagule dispersal in marine and terrestrial environments: a community perspective. *Ecology*, 84, 2007-2020.

Marleau, J.N., Guichard, F., Mallard, F., & Loreau, M. (2010). Nutrient flows between ecosystems can destabilize simple food chains. *Journal of Theoretical Biology*, 266, 162-174.

Nathan, R. (2006). Long-distance dispersal of plants. *Science*, 313, 786-788.

Schurr, F.M., Spiegel, O., Steinitz, O., & Trakhtenbrot, A. (2009). Long-distance seed dispersal. In: Annual Plant Reviews Volume 38: Fruit Development and Seed Dispersal. pp. 204-237.
